# Elucidating the role of carrier proteins in cytokine stabilization within double emulsion‐based polymeric nanoparticles

**DOI:** 10.1002/btm2.10722

**Published:** 2024-09-05

**Authors:** Emily R. Rhodes, Nicole B. Day, Emma C. Aldrich, C. Wyatt Shields, Kayla G. Sprenger

**Affiliations:** ^1^ Department of Chemical and Biological Engineering University of Colorado Boulder Boulder Colorado USA; ^2^ Biomedical Engineering Program University of Colorado Boulder Boulder Colorado USA

**Keywords:** cancer, cytokines, interfacial behavior, molecular dynamics, nanoparticles

## Abstract

Polymeric micro‐ and nanoparticles are useful vehicles for delivering cytokines to diseased tissues such as solid tumors. Double emulsion solvent evaporation is one of the most common techniques to formulate cytokines into vehicles made from hydrophobic polymers; however, the liquid–liquid interfaces formed during emulsification can greatly affect the stability and therapeutic performance of encapsulated cytokines. To develop more effective cytokine‐delivery systems, a clear molecular understanding of the interactions between relevant proteins and solvents used in the preparation of such particles is needed. We utilized an integrated computational and experimental approach for studying the governing mechanisms by which interleukin‐12 (IL‐12), a clinically relevant cytokine, is protected from denaturation by albumin, a common stabilizing protein, at an organic‐aqueous solvent interface formed during double emulsification. We investigated protein–protein interactions between human (h)IL‐12 and albumin and simulated these components in pure water, dichloromethane (DCM), and along a water/DCM interface to replicate the solvent regimes formed during double emulsification. We observed that (i) hIL‐12 experiences increased structural deviations near the water/DCM interface, and (ii) hIL‐12 structural deviations are reduced in the presence of albumin. Experimentally, we found that hIL‐12 bioactivity is retained when released from particles in which albumin is added to the aqueous phase in molar excess to hIL‐12 and sufficient time is allowed for albumin‐hIL‐12 binding. Findings from this work have implications in establishing design principles to enhance the stability of cytokines and other unstable proteins in particles formed by double emulsification for improved stability and therapeutic efficacy.

**Translational Impact Statement**

Cytokines are a useful class of therapeutic proteins. However, rapid denaturation limits their clinical use. Encapsulation of cytokines with carrier proteins in polymeric particles formed by double emulsification can confer stability, but the mechanisms are not well‐understood. By studying interleukin‐12, we identified two mechanisms by which albumin, a carrier protein, can impart stabilization. This knowledge may provide strategies for the design of drug delivery vehicles with improved capacity to stabilize cytokines.

## 1 INTRODUCTION

Cytokines have garnered significant attention as potential therapeutic agents for immune diseases due to their precise regulation of immune cell activity.^1^ However, their short half‐lives, resulting from both intrinsic instability and rapid *in vivo* degradation by proteolytic enzymes,^2^ present major challenges for drug delivery. In the case of interleukin (IL)‐12, a promising cytokine for cancer immunotherapy,^3^ high systemic doses are required to show therapeutic efficacy, resulting in strong off‐target effects and dose‐related toxicities.^4^, ^5^, ^6^ Thus, despite its well‐documented anti‐tumor effects in preclinical models^7^, ^8^ and therapeutic potential for other diseases,^9^, ^10^ its clinical utility has been limited. Encapsulation of IL‐12 and other cytokines into micro‐ and nanoparticle formulations offers promise for regulating spatiotemporal delivery,^7^ thereby reducing dosages and off‐target effects. However, techniques such as double emulsion solvent evaporation,^11^ commonly used for formulating cytokines into polymer nanocarriers, often compromise cytokine stability by exposing them to extreme temperatures, harsh solvents, and/or deleterious interfacial interactions.^4^ To address this challenge, polysaccharides (e.g., mannitol, trehalose), surfactants (e.g., polyvinyl alcohol [PVA]), and especially carrier proteins (e.g., albumin) are frequently used in the aqueous phase during emulsification to stabilize cytokines.^12^ However, the mechanisms governing interactions between these stabilizers and cytokines to promote cytokine stability remain unclear. Understanding these mechanisms is crucial for improving the stability and bioactivity of cytokines in drug‐delivery systems, paving the way for their broader therapeutic application.

In this study, we employed a combination of *in silico* and *in vitro* approaches to study the mechanisms underlying the protection of IL‐12, a clinically relevant cytokine, from denaturation during double emulsification. To promote clinical and translational relevance, we examined the formulation of human (h)IL‐12 in nanoparticles prepared from the FDA‐approved polymer poly(lactic‐co‐glycolic acid) (PLGA). Our objectives were twofold: first, to identify factors contributing to the instability of hIL‐12 during the double emulsification process typically used to formulate cytokines, and second, to elucidate the role of bovine serum albumin (BSA), a common carrier protein,^12^ in stabilizing hIL‐12. We hypothesized that BSA confers a stabilizing effect through two mechanisms: (i) by crowding at a water/dichloromethane (DCM) interface, thereby preventing hIL‐12 from contacting and denaturing at the interface, and (ii) through direct binding of one or more BSA molecules to hIL‐12 to stabilize its structure. Deciphering these mechanisms could inform the design and formulation of novel particles for more effective therapeutic delivery of IL‐12. Furthermore, this work establishes a framework to study the interactions between other unstable proteins (e.g., chemokines, enzymes, growth factors, cytokines) with therapeutic potential and stabilizers to improve their stability and biological activity.

## 2 RESULTS

### 2.1 PLGA nanoparticle formulation and characterization

Double emulsification is widely used to formulate proteins and/or other hydrophilic drugs into polymeric nanoparticles.^11^ Once the water‐in‐oil‐in‐water (W1/O/W2) emulsion is formed, the oil phase evaporates, leaving behind solid polymeric nanoparticles with pockets of protein and/or hydrophilic drug. To protect proteins in the hydrophilic W1 pockets, stabilizing agents such as polysaccharides, surfactants, and carrier proteins are added prior to the first emulsification step. Given the large number of variables in these systems, we constrained our focus to (i) formulations of hIL‐12, due to its clinical potential, (ii) PLGA as the base polymer to form the particles, due its clinical precedence, and (iii) BSA as a stabilizing agent, due to its low cost and widespread use. During particle preparation, parameters such as the ratio of lactic acid to glycolic acid, polymer molecular weight, and sonication intensity—which are often adjusted to achieve specific particle properties—were kept constant to isolate the effects of BSA on hIL‐12 only.

**FIGURE 1:**
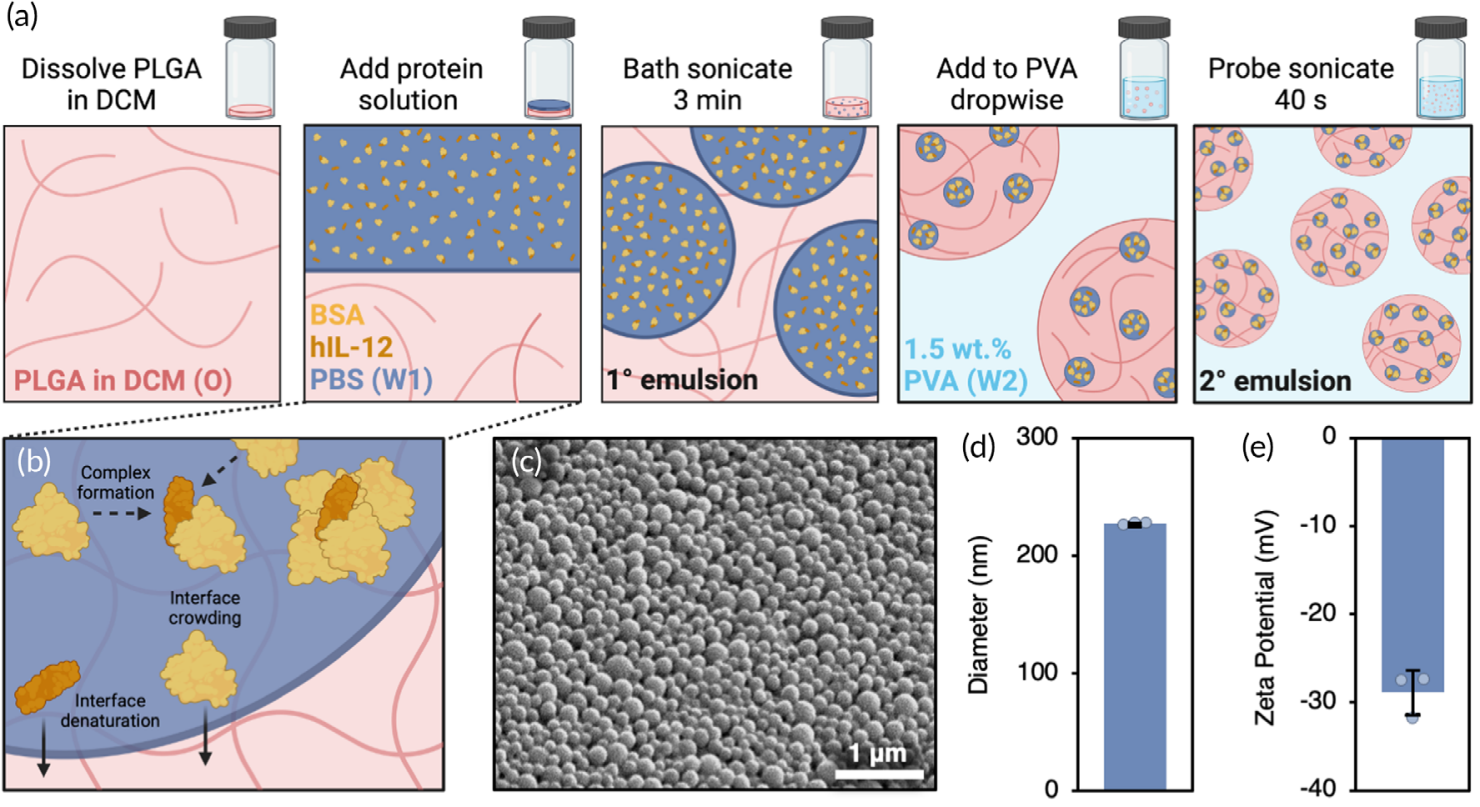
Double emulsion particle preparation and characterization. (a) Interfaces generated during preparation of PLGA nanoparticles containing hIL‐12 and BSA. (b) Hypothesized protein fate at the W1/O interface. (c) Representative SEM image of PLGA nanoparticles containing BSA and hIL‐12. (d) Average size and (e) zeta potential of experimental particles (*N* = 3 ± SD).

To support clinical and translational relevance, particles were prepared following established methods for double emulsion solvent evaporation.^11^ Briefly, proteins were dissolved in phosphate‐buffered saline (PBS) to form the W1 phase, which was then dispersed in the organic phase containing PLGA in DCM (O) to create the primary emulsion (1°). The secondary emulsion (2°) was then generated by dropwise addition to a 1.5 wt.% PVA solution (W2) to stabilize the colloidal dispersion, followed by sonication to produce nanoparticles (Figure 1a). Potential opportunities for loss of payload during cytokine encapsulation include formation of the W1/O interface (Figure 1b), transfer of the 1° emulsion to W2, and premature release from the particles during the overnight solvent evaporation step. Since the methodology of particle formulation remained consistent across batches, any observed changes in hIL‐12 release were solely attributed to destabilizing interfacial effects.

We performed scanning electron microscopy (SEM) (Figure 1c) and dynamic light scattering (DLS) to characterize the particles and ensure batch‐to‐batch consistency. The intensity‐weighted hydrodynamic diameter of the particles was not significantly affected by changes in BSA, resulting in an average particle size of 227 ± 1 nm between batches (Figure 1d), with an average polydispersity index of 12.1 ± 1.7% (Figure S1). Additionally, the zeta potential of the particles depended on PLGA chemistry, which remained constant across conditions (Figure 1e). Consequently, the comparison of hIL‐12 release from the particles was independent of particle properties.

### 2.2 Preferred distances and orientations of BSA and hIL‐12 near the W1/O interface

**FIGURE 2:**
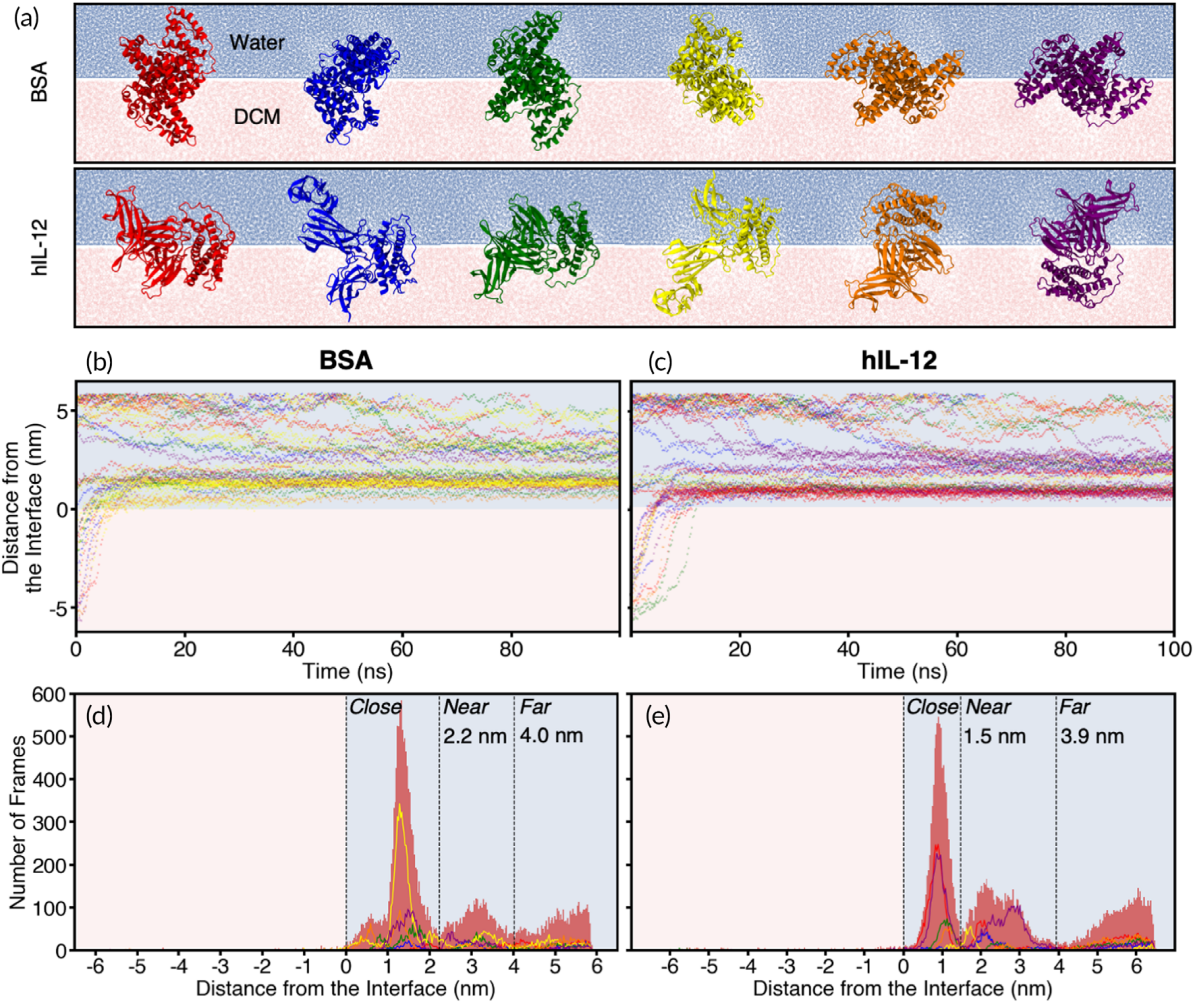
*In silico* characterization of BSA and hIL‐12 interfacial behaviors. (a) Initial molecular orientations of BSA (top row) and hIL‐12 (bottom row) relative to a W1/O interface. Distance of (a) BSA or (c) hIL‐12 from the interface for all simulations as a function of simulation time, colored according to the initial orientations in (a). Number of simulation frames capturing the indicated position of (d) BSA or (e) hIL‐12 as their positions equilibrated over 100 ns.

Molecular dynamics (MD) simulations were employed to evaluate the conformational dynamics of the proteins and their affinity for the W1/O interface, offering molecular‐level insights into cytokine behavior during the double emulsification process. A rectangular simulation box was solvated halfway with water (representing W1) and halfway with DCM (representing O) to establish the W1/O interface. To expose various protein surfaces to the interface, both hIL‐12 and BSA were initially positioned in six unique orientations relative to the interface, rotated 90° about the X and Y axes (Figure 2a). This process was repeated for seven different starting positions of each protein relative to the solvent positions: (1, 2) fully surrounded by each solvent (water or DCM); (3, 4) within 1.2 nm of the interface in each solvent; (5, 6) touching the interface in each solvent; and (7) equally spanning the interface between the two solvents. We note that the 1.2 nm cutoff distance, defined in our simulations, marks the point at which non‐bonded interactions, specifically van der Waals forces, between the proteins and the interface began to manifest, prompting dynamic responses from the proteins. Collectively, this approach allowed us to comprehensively characterize the equilibrium behavior of each protein at the interface across the 42 simulations.

Tracking the positions of hIL‐12 and BSA over time, we observed that both proteins migrated towards the interface regardless of their initial solvent placement, ultimately residing with their centers‐of‐mass (COM) exclusively situated in the water phase (Figure 2b,c). Nonetheless, their structures spanned the interface, leading to significant interfacial effects. This is evident in Supplemental Videos 1 and 2, where the density of each system component (water, DCM, BSA, and hIL‐12) is plotted along the orthogonal dimension of the simulation box over time, for two example simulations. Snapshots from the final frames of the videos (Figure S2a,b) demonstrate that both BSA and hIL‐12 concentrate at the interface, exhibiting a peak in density distribution that aligns close to the interface—approximately where DCM and water densities converge—yet remains primarily situated in the water phase. Precise interface location calculations are provided in Section 4.3.

Interestingly, proteins originating from the water phase tended to equilibrate with their COMs positioned farther away from the interface compared to those originating from the DCM phase (Figure 2b,c). Specifically, when BSA originated from the DCM and water phases, it equilibrated with its COM positioned 1.4 and 3.0 nm from the interface on the water side, respectively (Figure S3a,c). Conversely, hIL‐12, originating from these same phases, equilibrated with its COM positioned 1.0 and 2.8 nm from the interface on the water side, respectively (Figure S3b,d). The presence of two distinct distance preferences for each protein suggests that fully partitioning out of the DCM phase to reside at the interface on the water side may pose a challenge, leading them to remain closer to the interface when originating from the DCM phase. These results suggest that adding stabilizing agents to the oil phase that exhibit similar solubility in aqueous and organic solutions could result in tighter coupling along the W1/O interface, providing an additional barrier to prevent cytokines from adsorbing to the interface from the W1 phase. Potential strategies for interfacial crowding are described in more detail in Section 3.

We observed that in simulations where the proteins were initially positioned in the water phase far from the interface (i.e., fully surrounded by solvent), the onset of movement towards the interface was largely stochastic. Consequently, reaching the interface did not always occur within the simulated timeframe (Figure 2b,c). In contrast, simulations where the proteins were initially positioned in DCM consistently showed rapid movement towards the interface. This strong drive towards the interface, particularly the water side of the interface, was likely facilitated by increased hydrogen bonding capabilities between the proteins and water compared to DCM (Figure S4a,b).

At the interface, in addition to displaying preferred interfacial COM distances, the proteins also exhibited distinct orientational preferences, with their initial orientations, depicted in Figure 2a, dynamically evolving throughout the simulations. To rigorously analyze these orientations, we colored the datapoints in every frame of the simulations according to the closest orientation. This involved calculating the deviation of every protein atom from its reference position in each orientation, ultimately selecting the orientation with the smallest measured deviation. We observed that orientations most similar to the yellow initial orientation (i.e., “yellow‐like” orientations) were overwhelmingly the most frequently adopted by BSA at an interfacial distance of 1.4 nm (Figure 2d), trailed by purple‐ and orange‐like orientations. Notably, orientations most similar to the red, green, and blue initial orientations were least frequently adopted by BSA at the interface. Orientational preferences of BSA at the interface may stem from differences in how its α‐helices—which comprise most of its structure—orient with respect to the interface. Indeed, the α‐helices in the yellow initial orientation in Figure 2a are oriented most parallel to the interface on average, followed by the purple and orange initial orientations, and finally the red and green initial orientations. The yellow initial orientation allows for the maximum number of residues and the largest surface area of BSA to interact with the interface compared to the other orientations (Table S1). This preference for a parallel interfacial orientation aligns with the notion that proteins spread at interfaces and form a coating due to surface tension, established in past studies^13^, ^14^, ^15^, ^16^, ^17^ and corroborated through in‐tandem dynamic tension and X‐ray reflectivity studies for antibodies.^18^ This surface tension, which acts parallel to the interface, actively positions and maintains proteins in specific orientations.

To better understand the relevance of surface tension in our simulations, we calculated the average interfacial surface tension (see Section 4.4) in the presence of either BSA or hIL‐12 at the interface and in the presence of each protein at the interface separately (Figure S5). Interestingly, we observed that the surface tension was slightly higher in the presence of BSA compared to hIL‐12. Subsequently, we applied the Girifalco–Good equation to determine the interaction parameter, ϕ.^19^ In each case, we found ϕ to be within the experimental range for non‐associated liquids such as DCM and water (0.5–0.8, assuming the surface tension for water is 720 [bar⋅nm] and DCM is 280 [bar⋅nm]),^20^ validating the observed effects of interfacial surface tension in our systems.

At the preferred interfacial COM distance of 0.8 nm, hIL‐12 was observed to primarily adopt orientations most closely resembling the red and purple initial orientations in Figure 2a, followed by green and orange‐like orientations (Figure 2e). Blue and yellow‐like orientations were almost never adopted by hIL‐12 at this interfacial distance. In these blue and yellow‐like orientations, the longest axis of hIL‐12 appeared to align almost perpendicular to the interface, with the flexible protruding “tip” of hIL‐12 residing in bulk solvent. Similar to the vertically aligned α‐helices of BSA, these more vertically aligned hIL‐12 orientations increase the surface area of the protein exposed to interfacial forces. Surface tension is likely responsible for affecting the orientation of hIL‐12 to maximize the number of residues and total surface area aligned with the interface. This is illustrated in Table S1, where the purple initial orientation of hIL‐12 shows the largest interface‐accessible surface area. Interestingly, the red initial orientation displays a relatively low interface‐accessible surface area, suggesting the six coarse‐grained initial orientations in Figure 2a cannot entirely capture the details of the orientational preferences of hIL‐12 at the interface.

To better capture the effects of protein spreading at the interface beyond what was possible by comparing to the coarse‐grained initial orientations, we reanalyzed the simulation data from Supplemental Videos 1 and 2, calculating the width of the density distribution for each protein at each time point. We then plotted this data on a single graph, coloring the points by time to illustrate changes in protein alignment and spreading at the interface (Figure S6). The results indicate a narrowing of the width of the density distributions for each protein in the direction perpendicular to the interface, supporting our earlier observations of interfacial spreading. This suggests that, while all proteins align and spread along an interface, the density of globular proteins like BSA do not narrow to the same extent at the interface. This provides a barrier to protect other, more vulnerable proteins, like IL‐12, from interfacial forces.

**FIGURE 3:**
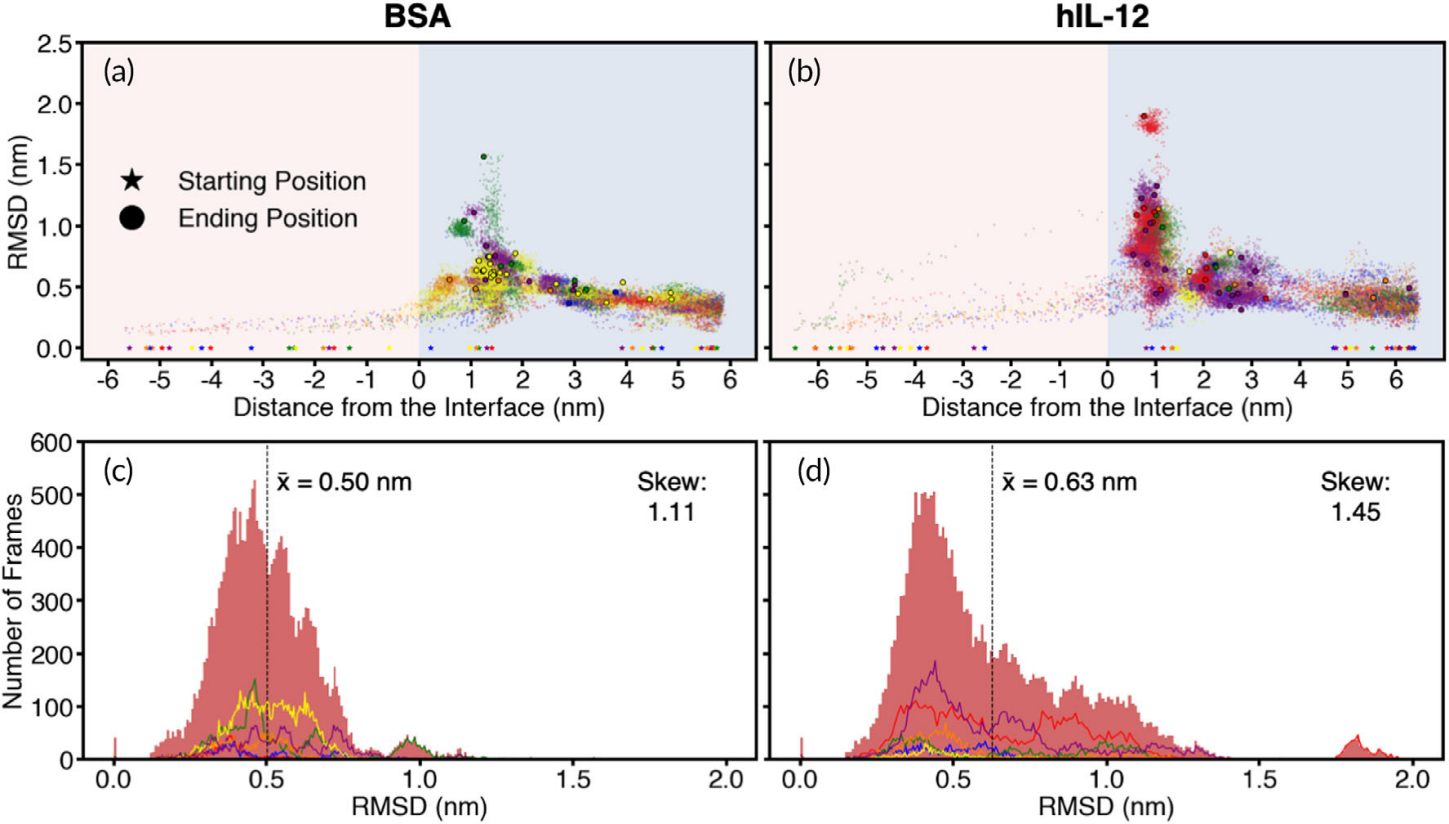
Molecular deviations across simulations. RMSD of (a) BSA and (b) hIL‐12 as their positions relative to the W1/O interface change. Stars and black circles indicate the starting and ending positions for each simulation, respectively. Distribution of RMSD values of (c) BSA and (d) hIL‐12 across all trajectories, with the median RMSD shown by dashed vertical lines. Colors correspond to the initial orientations described in Figure 2a.

To detect structural changes of the proteins during simulations, we monitored the root‐mean‐square deviation (RMSD) of the proteins (Figure 3; see Figure S7 for raw data). Both BSA and hIL‐12 exhibited elevated RMSDs, evidence of structural changes, near the interface compared to their initial configurations (Figure 3a,b). However, the increase in RMSD was more pronounced for hIL‐12 than BSA, as quantified in Figure 3c,d, indicating a greater vulnerability of hIL‐12 to interface‐induced structural changes. Notably, specific orientations were observed to contribute most prominently to the increase in interfacial RMSD of each protein. For BSA, the greatest increases in interfacial RMSD were observed for green‐like orientations. The near‐perpendicular alignment of the green initial orientation relative to the interface results in surface tension concentrated on a relatively small surface area exposed to the interface, leading to heightened structural changes and increases in RMSD. We note that orientations most similar to the red initial orientation were rarely adopted at the interface by BSA (Figure 2d), resulting in limited interfacial RMSD data for red‐like orientations. Furthermore, yellow‐like orientations, which were most frequently observed at the interface and comprised the most parallel‐oriented α‐helices with respect to the interface, exhibited a relatively high resistance to interface‐induced structural changes (Figure 3c). Orientations most similar to the blue orientation were again seldom adopted at the interface by BSA (Figure 2d), resulting in limited interfacial RMSD data for blue‐like orientations.

In contrast to BSA, where orientations with relatively high resistance to interface‐induced structural changes were found to be adopted most frequently at the interface, hIL‐12 exhibited the opposite trends. Specifically, for hIL‐12 (Figure 3b,d), the greatest interfacial RMSD increases were observed for purple and red‐like orientations, which were also most frequently adopted at the interface (Figure 2e). Notably, orientations of hIL‐12 most closely resembling the blue and yellow initial orientations were rarely observed at the interface, thus limiting the conclusions that can be drawn between interfacial RMSD and orientation in these cases.

In summary, these findings provide preliminary indications that hIL‐12 is more susceptible to interfacial‐induced structural changes compared to BSA. This highlights the potential for optimizing the use of BSA as a shield to protect hIL‐12 against these interfacial effects, a concept we explore further in later sections. Overall, for one protein to provide protection to another at the interface, the carrier protein must be resistant to forces acting parallel to the plane of its largest surface area. Our simulations reveal that the elongated structure of hIL‐12 leads to significant vulnerabilities at the W1/O interface, while the globular, α‐helical structure of BSA exhibits robust strength against interfacial tension. Thus, these results help explain why BSA is a suitable carrier protein in particles made by double emulsion solvent evaporation.

### 2.3 Conformational dynamics of BSA and hIL‐12 near the W1/O interface

**FIGURE 4:**
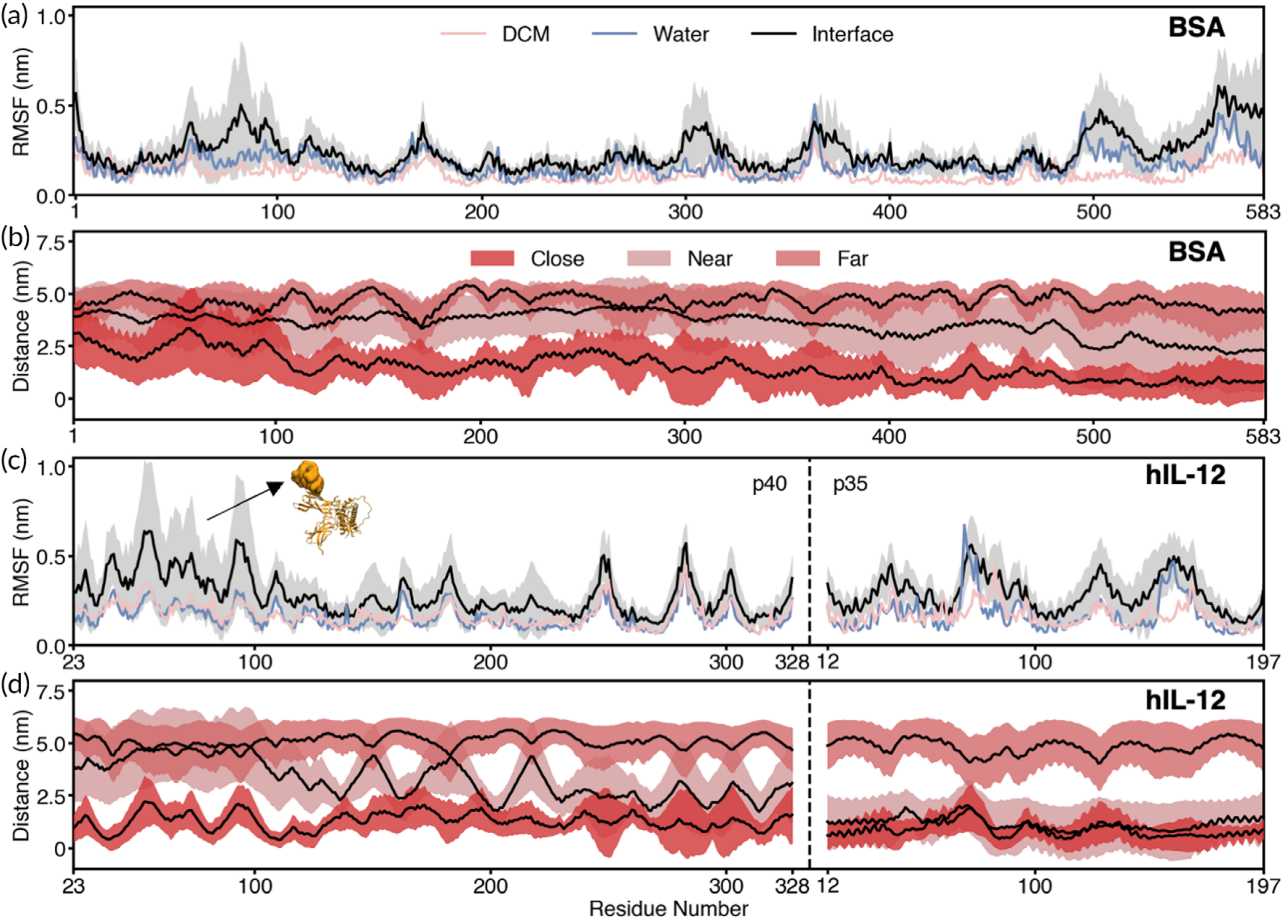
Per residue interfacial interactions by BSA and hIL‐12. (a) RMSF values for BSA in different solvent conditions. Gray shadows represent standard deviation of the interface (black line) condition across previously described interface simulations (*N* = 42). (b) Average distance between the interface and each BSA residue for the distance categories shown in Figure 2b. Shaded regions represent standard deviation for each regime. (c) RMSF values for hIL‐12 in different solvent conditions. Protein structure inlay highlights residues 23–113 on the hIL‐12 molecule. (d) Average distance between the interface and each hIL‐12 residue for the distance categories shown in Figure 2e.

To delve deeper into the impacts of the W1/O interface on the conformational dynamics of the proteins, we computed the average root‐mean‐square fluctuation (RMSF) of BSA and hIL‐12 residues, analyzed in relation to interfacial distance. We found that both BSA and hIL‐12 residues exhibited broadly similar RMSF values in pure water and pure DCM (Figure 4a,c, blue and pink lines, respectively). These results suggest that DCM is not the primary factor contributing to conformational instability in the proteins during the double emulsion process over the timescales associated with our simulations.

In simulations where an interface was present (Figure 4a,c, black lines), the RMSF of the protein was calculated over the last 50 ns of the simulation and averaged across all protein‐interface distance regimes defined in Figure 2d,e. Any differences in the behavior of the proteins in the simulations with an interface versus in pure water or pure DCM were thus directly attributed to interfacial effects. In this context, we observed that both proteins exhibited higher fluctuations overall in the presence of the W1/O interface compared to in either pure solvent, with this effect being more pronounced for hIL‐12. Specifically, although a few small regions of BSA showed notably higher RMSF values in the presence of the interface, the overall increase in residue‐level fluctuations of BSA at the interface compared to those in either pure solvent was small (Figure 4a). However, for hIL‐12, we observed markedly higher residue‐level fluctuations across large regions of the protein in the presence of the interface compared to in either pure solvent (Figure 4c). This difference was most pronounced for the p40 subunit (chain A) of hIL‐12, where the largest RMSF differences between the interface and pure solvent occurred for residues 23–113. Notably, these residues correspond to the aforementioned “tip” region of hIL‐12 (Figure 4c, inset image).

To gain additional insights into the ability of the W1/O interface to perturb the dynamics of residues in hIL‐12, we isolated all frames from each of the three protein‐interface distance regimes (close, near, and far, as labeled in Figure 2d,e) and calculated the distance of each protein residue from the interface, averaging the values across the respective frames in each regime. We performed the same analysis for BSA as a control. For both hIL‐12 and BSA, we observed a largely consistent average interfacial distance across all residues in the “far” regimes, as expected, indicating no preferential orientation in the bulk solvent (Figure 4b,d, respectively). Similar behavior was observed for BSA as it approached the interface in the “near” regime, aligning with the findings in Figure 2d. However, in the “close” regime, BSA residues 1–100 exhibited a larger average interfacial distance compared to the rest of the protein, suggesting a tendency for these residues to orient away from the interface. Figure S8a illustrates how the yellow‐like orientations of BSA, specifically position residues 0–100, are furthest from the interface. Thus, these observations corroborate the results depicted in Figure 2d, which as previously discussed, show the predominance of yellow‐like BSA orientations at the interface.

In contrast to BSA, hIL‐12 exhibited an inconsistent average interfacial distance across all residues as it approached the interface in the “near” regime, indicating an orientational dependence (Figure 4d), as observed in Figure 2e. Specifically, in this regime, the p35 subunit (chain B) tended to orient towards the interface, shown by average interfacial distance values comparable to those in the “close” regime. Conversely, the tip of the p40 subunit generally oriented away from the interface, with average interfacial distance values similar to those in the “far” regime. Upon reaching the interface and entering the “close” regime, hIL‐12 reorients, aligning residues 23–113 (the tip region) parallel to the interface. Figure S8b illustrates how the purple‐like orientations of hIL‐12, and to a lesser degree the red‐like orientations, specifically position residues 23–113, are far from the interface, consistent with the predominance of these orientations observed in Figure 2e.

To determine which distance regime/hIL‐12 tip orientation contributes most to the RMSF increases seen in Figure 4c, we compared the above results with those in Figure 2b, which shows a relatively low RMSD for hIL‐12 in the “near” regime compared to the “close” regime. This suggests that the observed increases in RMSF primarily occur when the tip region of hIL‐12 is oriented parallel the W1/O interface in the “close” regime. Given the highly flexible and protruding nature of the hIL‐12 tip region, it is perhaps unsurprising that it experiences the greatest increases in conformational dynamics at the interface due to heightened vulnerability to interfacial surface tension. Overall, these findings offer additional evidence suggesting that hIL‐12 may be more prone to structural and dynamical alterations induced by interfacial effects compared to BSA. Furthermore, the results highlight the tip region of hIL‐12 as particularly vulnerable to these interfacial effects, implying that stabilizing this region could enhance the bioactivity of hIL‐12 during emulsification. Strategies for improving the stabilization of cytokines using this insight are described in Section 3.

### 2.4 Molecular docking of BSA to hIL‐12 to assess impacts of protein–protein interactions on interfacial behavior

To more directly explore the potential of BSA to act as a stabilizer and protect hIL‐12 from interfacial effects, we performed a molecular docking study using ClusPro, a rigid body docking server.^21^ Given the lack of experimentally resolved structures of their interaction complex, this step was essential for predicting binding modes between BSA and hIL‐12 and provided realistic starting configurations for our subsequent MD simulations.

**FIGURE 5:**
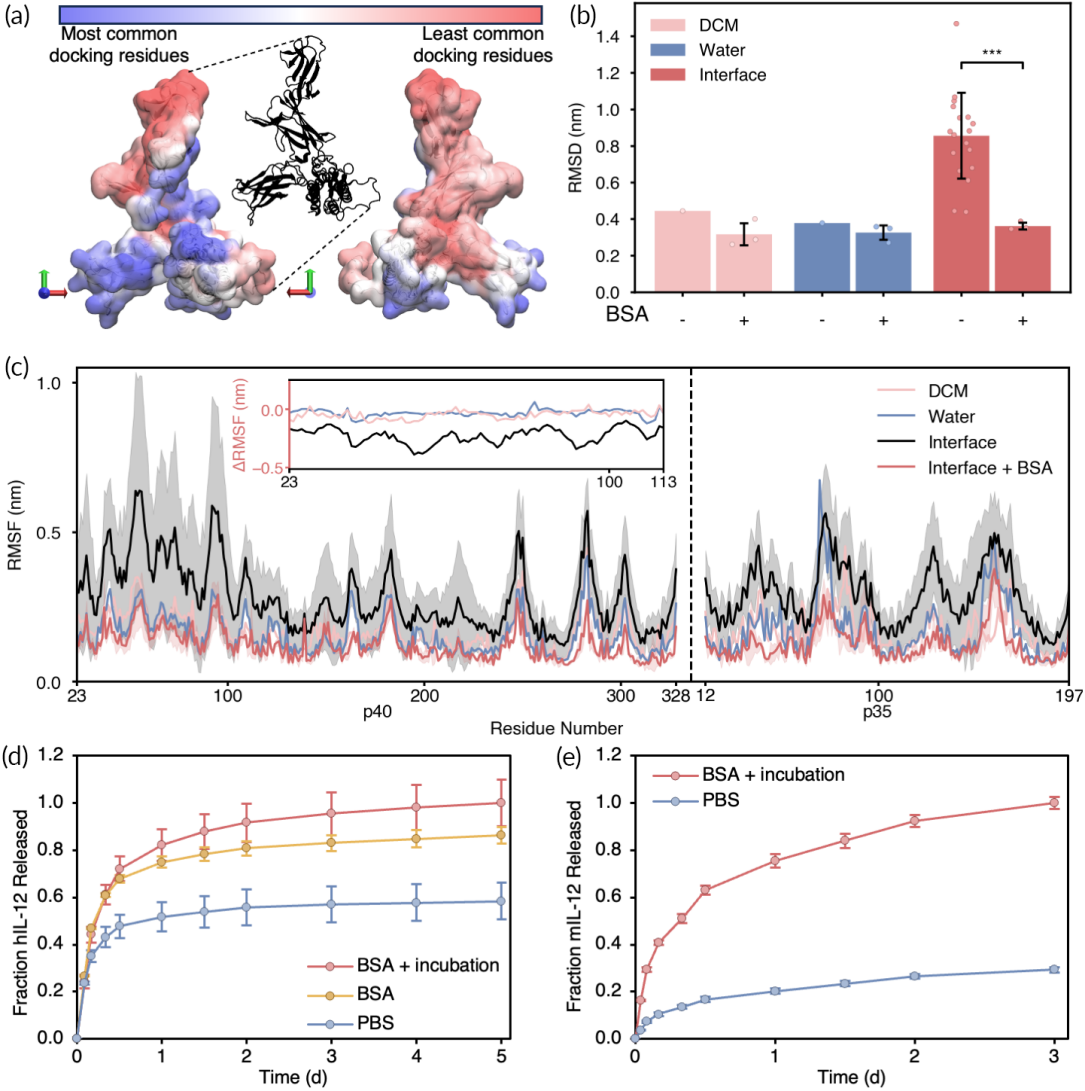
BSA stabilization of hIL‐12. (a) BSA docking frequency to hIL‐12 (shown from two angles) by region. (b) Average RMSD values (over the last 50 ns) for hIL‐12 with or without docked BSA in different solvent conditions (*N* = 3 ± SD). The interface average is calculated for all RMSD values “close” to the interface. (c) RMSF of hIL‐12 in various environments. Shaded regions represent standard deviation for the Interface (*N* = 42) and Interface + BSA conditions (*N* = 3). Inset: change in RMSF between the Interface + BSA condition and all others (without BSA) within the key residue region. (d) hIL‐12 released from particles with various stabilizing conditions over time. (e) mIL‐12 released from particles with the optimal stabilizing formulation.

One molecule of BSA was docked to each of the 11 structures of hIL‐12 isolated from the pure water simulation every 10 ns from 0 to 100 ns. In each case, we selected the structure at the center of the most populated cluster, representing the BSA‐hIL‐12 complex with the lowest RMSD to all other complexes within that cluster, thus making it the most typical structure. These center structures were then used in subsequent docking calculations. Additionally, we retained the center structures from the 10 most populated clusters for further analysis, specifically for the construction of Figure 5a, introduced below. To each of the 11 BSA‐hIL‐12 complexes from the most populated cluster, we docked one additional molecule of BSA, repeating the process of setting aside the center complex from the most populated cluster for further docking calculations and retaining additional complexes for analysis. This process continued until four BSA molecules were bound to each of the 11 hIL‐12 structures isolated from the pure water simulation trajectory. Beyond this point, additional BSA molecules docked to other BSA molecules rather than to hIL‐12; therefore, we considered a ratio beyond 4:1 BSA to hIL‐12 molecules as molar excess.

By characterizing the frequency with which hIL‐12 residues were in contact with (i.e., within 5 Å of) BSA residues across all retained BSA‐hIL‐12 complexes (*N* = 440 total models), we observed that BSA preferentially docks to and interacts with the α‐helical domain and proximal *β*‐sheet region of hIL‐12 (Figure 5a). Importantly, we also found that the region of hIL‐12 with the least interaction with BSA is the flexible, protruding tip, previously identified as the region most susceptible to interface‐induced stresses (Figure 4a). This preliminary finding suggests that the lack of direct, frequent interaction between the tip of hIL‐12 and BSA may lead to heightened exposure of this region to interfacial forces during emulsification, resulting in altered hIL‐12 conformational dynamics through increased residue‐level fluctuations.

To further explore this hypothesis, we performed additional 100 ns MD simulations on selected docked complexes and compared them to simulations of hIL‐12 alone in comparable conditions. Specifically, we simulated three docked complexes each of hIL‐12 bound to one, two, three, and four BSA molecules, with each complex solvated in (i) pure water and (ii) pure DCM. For the complex with four BSA molecules bound, we also performed a simulation where the complex was initially placed at (iii) the W1/O interface. We then computed the RMSD of hIL‐12 in each complex, averaging the results across the last 50 ns of the three simulations with the same solvent conditions and number of BSA molecules bound. The results are shown in Figure 5b for hIL‐12 alone and bound by four BSA molecules, and in Figure S9 for the remaining complexes. As described previously, we observed a significant spike in the RMSD of hIL‐12 alone in the presence of the interface compared to in either pure solvent (Figure 5b). With four BSA molecules bound, we observed minor reductions in the RMSD of hIL‐12 in pure water and pure DCM, and a substantial reduction in the RMSD of hIL‐12 at the W1/O interface to similar levels in all three cases.

We also monitored the RMSF of hIL‐12 in these different environments. In Figure 5c, we present the RMSF of hIL‐12 isolated from complexes with four BSA molecules bound at the W1/O interface, contrasting the results to that of hIL‐12 alone at the interface, in pure DCM, and in pure water (as shown in Figure 4c), with additional simulations shown in Figure S10a–e. As discussed earlier, in the absence of BSA, hIL‐12 displayed increased fluctuations at the interface compared to in either pure solvent, especially for the tip region (residues 23–113). Notably, with four bound BSA molecules, the RMSF of hIL‐12 at the W1/O interface generally decreased across all residues, particularly in the tip region (the change in RMSF with the addition of BSA is shown in the inset), returning to levels observed in pure solvent (Figure 5c).

Overall, these results substantiate the notion that the direct binding of BSA to the core domains of hIL‐12 enhances the stability of its entire structure, including the vulnerable tip region. This stability mitigates interface‐induced deviations in the conformational dynamics of hIL‐12, particularly evident in the tip region. Furthermore, these results suggest that pre‐incubating hIL‐12 with BSA before the first emulsification, allowing for sufficient direct binding to occur, may be effective in preventing the interface from exerting destabilizing effects on hIL‐12. We investigated this hypothesis in experiments detailed in the following section.

### 2.5 Impacts of pre‐incubation time on hIL‐12 release from BSA‐loaded particles

To validate the computational findings that BSA docking to hIL‐12 imparts stability at the interface, we formulated PLGA double emulsion particles containing BSA in molar excess (i.e., >4:1 BSA:hIL‐12) either with or without incubation of the proteins in the W1 phase (i.e., prior to the first emulsification step) to allow for complete protein–protein binding. We estimated that any binding that was to occur would take place within 10 min of incubation due to diffusion timescales of the proteins in 80 μl of PBS. On the other hand, sequential addition of BSA and hIL‐12 into DCM to create the primary emulsion (i.e., no incubation) would likely result in limited protein–protein binding during formation of the primary emulsion, therefore isolating stabilizing effects due to interfacial crowding only. Measurement of hIL‐12 loading and stabilization in particles was determined through controlled release studies. hIL‐12 was measured using a monoclonal enzyme‐linked immunosorbent assay (ELISA) with a polyclonal detection antibody. After 5 days, hIL‐12 release was normalized by sample volume and compared against the highest cumulative release of any condition.

Particles formulated with pre‐incubated hIL‐12 and BSA in excess—representing hIL‐12 stabilization by both docking and interface crowding—resulted in the highest cumulative release of hIL‐12 (Figure 5d). BSA‐loaded particles without incubation released, and therefore stabilized, 86.2% of that amount, while control particles without BSA only released 58.5% of the maximum released hIL‐12. This confirms our computational findings that hIL‐12 encapsulated in particles prepared by double emulsification have substantially less structural unfolding when stabilized by BSA. Furthermore, increased detection of hIL‐12 from BSA‐loaded particles with incubation compared to BSA‐loaded particles without incubation suggests that the two hypothesized mechanisms of stabilization may work in concert to prevent hIL‐12 denaturation at the interface by multiple methods; steric hindrance at the interface by unbound BSA reduces exposure of hIL‐12 to the interface, and hIL‐12 that is exposed to the interface is protected by the binding of BSA molecules. However, while both BSA and BSA + Incubation conditions showed significantly greater preservation of hIL‐12 by Day 5 than PBS (*p* = 0.00460 and *p* = 0.00458, respectively), there is no significant difference between the two BSA conditions (*p* = 0.0861). This suggests that steric hindrance may play a greater role in preventing hIL‐12 denaturation compared to direct binding when the carrier protein is in sufficient excess, or that the two mechanisms cannot be completely decoupled using this approach.

While we focused on human IL‐12 for modeling and most of the experiments, murine models are still used routinely for drug delivery system studies. Importantly, the IL‐12 p35 (α) subunit shares only 60% identity between mice and humans (Figures S11 and S12); as such, we performed an additional release study using murine IL‐12 (mIL‐12) to determine whether BSA‐mediated stabilization was useful in both systems. Using the highest (optimal stabilization; BSA in excess with pre‐incubation) and lowest (negative control; no BSA) encapsulation efficiency conditions from the previous study, we found that mIL‐12 incubated with BSA behaves similarly to hIL‐12, resulting in substantially higher release compared to the PBS control (Figure 5e). This suggests that BSA binding may be sufficiently consistent across the two species to impart protection to mIL‐12 structure in the same manner as hIL‐12. Additionally, surface crowding by BSA is likely agnostic to the type of cytokine.

### 2.6 hIL‐12 bioactivity to confirm hIL‐12 stabilization

**FIGURE 6:**
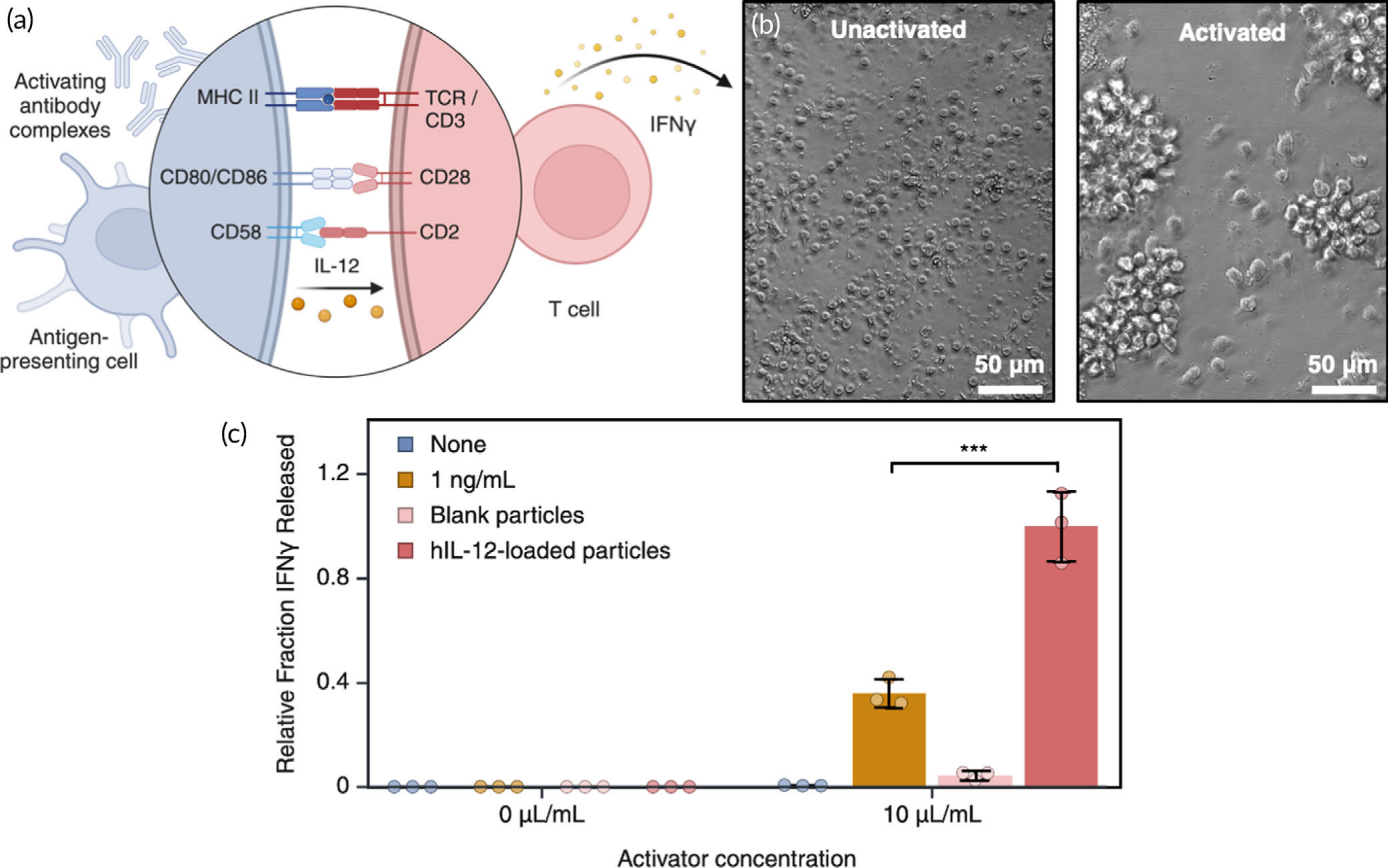
T cell activation by hIL‐12. (a) Graphical illustration of T cell activation pathways by antibody complexes (*in vitro*) or by APCs (physiologically) and IL‐12. (b) Bright‐field images of inactivated and activated PBMCs in culture. (c) Fraction of IFNγ release from PBMCs, as a measure of hIL‐12 bioactivity, relative to the maximum mass released by hIL‐12‐loaded particles (*N* = 3 ± SD).

To validate that hIL‐12 release from particles by ELISA is indicative of molecular bioactivity, we performed an *in vitro* study to measure cellular responses to hIL‐12‐containing particles. Biologically active IL‐12 binds to its high‐affinity receptor, IL‐12R, on the surfaces of T cells, natural killer cells, and dendritic cells.^22^ In response to IL‐12 stimulation, activated T cells secrete interferon (IFN)γ in an IL‐12‐dependent manner to propagate the inflammatory signal (Figure 6a).^23^ We therefore isolated human peripheral blood mononuclear cells (PBMCs) and measured the mass of secreted IFNγ in response to a commercial activating antibody complex and hIL‐12 after 24 h. Free hIL‐12 was used at a concentration of 1 ng/ml, and particles were added to reach the equivalent concentration of hIL‐12 after 24 h according to the release shown in Figure 5d; we used only the optimally stabilized particle condition (BSA + incubation) to validate bioactivity. We observed that activated PBMCs, which form clumps characteristic of activated T cells (Figure 6b), released negligible quantities of IFNγ without stimulation by hIL‐12 (Figure 6c). Furthermore, inactivated PBMCs did not release a detectable concentration of IFNγ, despite the addition of hIL‐12. In contrast, significant IFNγ was secreted by PBMCs incubated with particles loaded with hIL‐12, using the optimal formulation represented in Figure 5d (BSA in excess with pre‐incubation). In addition, the estimated 1 ng/ml of hIL‐12 release from the particles in 24 h outperformed a bolus addition of free hIL‐12 at the same concentration (*p* = 0.00155), demonstrating that sustained release of agonist can produce a greater response than a single, bolus addition. While blank particles (containing BSA but no hIL‐12) showed a minor stimulatory effect, the resulting secretion of IFNγ was more than 20× smaller than that of particles containing hIL‐12. Therefore, release of IFNγ in response to stimulation by hIL‐12‐containing particles indicates that the hIL‐12 active site is not inhibited by BSA binding nor does it unfold, and therefore the encapsulation and release of hIL‐12 from our particles retains cytokine bioactivity.

## 3 DISCUSSION

Particle‐based drug delivery offers key advantages over systemic administration of free drugs. Formulating particles with application‐specific functionality can enable spatiotemporal control over drug administration through controlled release and targeting, reducing adverse effects associated with high dosages or off‐target tissue damage.^24^ For unstable biologics such as cytokines, chemokines, enzymes, and growth factors, payload protection and extended circulation are particularly beneficial. A fundamental understanding of the mechanisms by which cytokine stabilization or destabilization occurs is necessary to identify effective stabilizing agents. As such, to study cytokine denaturation during emulsification, we used the simple, translatable case of particles made from the FDA‐approved polymer PLGA, encasing human IL‐12 (hIL‐12) with the carrier protein BSA. We found that this previously described nanoparticle fabrication method produced replicable particle batches with and without BSA,^12^ as determined by average diameter, size polydispersity, and zeta potential. However, the process of double emulsification presents a clear opportunity for hIL‐12 denaturation during the formation of the W1/O interface. Therefore, we conducted MD simulations to examine protein structural deviations in the presence of a W1/O interface and subsequently assessed the stabilizing effect of BSA on hIL‐12. While the interaction of BSA with the interface is not of primary concern in particle design and development, studying its interactions with the interface may elucidate structure‐based stabilizing features.

Using six different protein orientations, relative to their centers‐of‐mass, and seven different starting locations, relative to the W1/O interface, we found that both proteins moved rapidly to the water phase and positioned themselves at two primary distances from the interface, largely dependent on the solvent phase from which they originated. The cumulative number of MD simulation trajectory frames at each position indicated that BSA and hIL‐12 position preferentially close to the interface (BSA <2.2 nm, hIL‐12 <1.5 nm), despite the RMSD being at its highest in that position. Notably, hIL‐12 has a higher maximum and a higher average RMSD compared to BSA, indicating relatively less structural stability. A high RMSD suggests that a molecule may be beginning to denature; therefore, these results confirm that hIL‐12 requires stabilization when formulated in particles made with double emulsification due to its instability at the interface. Furthermore, the mean RMSD of BSA at 0.5 nm suggests that there is limited unfolding. While BSA is a larger molecule than hIL‐12, and therefore takes up more space at the interface, an improved stabilizing molecule may be one that shields cytokine from the interface to a greater degree, such as through increased size at the interface. Additionally, the stabilizing molecule should be resistant to forces acting parallel to the face of the protein with the largest, flattest surface area. To evaluate the potential of an alternate stabilizing molecule to protect a payload from the interface, we propose simulating changes in protein alignment and spreading at the interface (such as in Figure S6).

We additionally investigated residue‐specific fluctuations, again using BSA as a reference molecule for hIL‐12. BSA displayed limited fluctuation of residues at the interface compared to when it was in pure water or DCM, reiterating its lack of substantial structural changes. However, relatively large fluctuations at residues 23–113 in the p40 subunit of hIL‐12 suggest that this key region is responsible for structural changes observed in the cytokine. Distance from the interface of this region and of the p35 subunit demonstrate the way hIL‐12 reorients when interacting with the interface. Holistically, the density width of hIL‐12 narrows as it approaches the interface (i.e., extending parallel to the interface and shrinking perpendicular to the interface), placing the largest, flattest surface of the protein parallel to the interface. For alternate protein payloads, similarly measuring the RMSF and identifying unstable regions with large fluctuations may implicate a specialized carrier molecule that docks to the key region more strongly than BSA.

Next, we investigated the role of BSA in stabilizing hIL‐12. We found that the hIL‐12 RMSD was similar in both water and DCM and was reduced minimally when four BSA molecules were docked to it. We hypothesize that this reduction in RMSD is due to physical/steric limitations due to BSA binding, rather than protection of unstable regions. Interestingly, we saw that the RMSD of hIL‐12 alone at the interface was substantially higher than that in DCM; indeed, the presence of an interface rather than the properties of DCM itself causes structural instability in hIL‐12. This is also validated by the RMSF of hIL‐12 in water and DCM, which had negligible differences compared to the increase at the interface. However, when four BSA molecules were docked, the interface‐induced hIL‐12 RMSD increase was dampened to levels consistent with RMSD values associated with hIL‐12 in water and DCM. This analysis demonstrates that there is a substantial stabilizing effect caused by direct binding of four BSA molecules to one hIL‐12 molecule. A similar analysis of (i) carrier molecule docking to an alternate protein payload, to determine the minimum molar ratio for protection, and (ii) RMSD comparisons of the payload and the complex at the interface, to determine the degree of protection that docking may provide, would be advantageous for modified systems.

As the timescale of identifying a converged RMSD is much shorter than that of DCM solvent evaporation during particle formulation, we validated the observed trends by performing experiments to release hIL‐12 from PLGA particles. Cytokine released from particles without BSA represents hIL‐12 that escaped denaturation without protection; cytokine released from particles with BSA but without incubation represents hIL‐12 that was protected by interfacial crowding alone; cytokine released from particles with pre‐incubated BSA represents hIL‐12 that was protected by protein–protein binding and interfacial crowding together. Importantly, we observed that both mechanisms contributed to the improved stability of hIL‐12. The relative improvement of hIL‐12 release, and therefore protection, was greater with BSA pre‐incubation than without (1.7‐fold and 1.5‐fold, respectively). However, it is likely that BSA‐hIL‐12 binding still occurred without incubation within the primary emulsion, and as such, the two mechanisms cannot be completely decoupled during experiments. Therefore, while it appears that interfacial crowding has a greater contribution to hIL‐12 stabilization than protein–protein binding in these experiments, we hypothesize that the combination of stabilizing methods will still surpass that of one mechanism alone. We therefore recommend that particle cytokine carriers utilize a stabilizing molecule in excess and allow incubation time prior to the first emulsification to achieve the optimal payload stabilization by both mechanisms.

While IL‐12 has clear clinical potential due to its anti‐tumoral effects, especially when used in combination with other therapeutic modalities, most experimental studies still involve mouse models.^25^, ^26^ Murine IL‐12 acts on both murine and human IL‐12 receptors (IL‐12R), while human IL‐12 only interacts with human IL‐12R. Despite their structural differences, however, we found that the optimal particle formulation from our hIL‐12 release studies translated to a 3.4‐fold increase in stabilized mIL‐12. Future work must consider the species‐specific structures of these proteins when designing particle drug carriers for clinical translation that rely on stabilization via protein–protein binding.

Based on these findings, we propose that the protection of cytokine in particles prepared by double emulsion solvent evaporation could be further improved by (i) increasing the affinity of the carrier protein for the W1/O interface, (ii) reducing the affinity of the cytokine for the W1/O interface, and (iii) increasing the interaction strength between the carrier protein and cytokine, especially in molar excess, to ensure that more cytokine molecules are bound to carrier proteins. Improving the binding strength of cytokines with stabilizing molecules may have a limited effect when proteins are pre‐incubated, as we assume that complete binding can occur in that time. Additionally, if protein binding to the carrier protein is too strong, activity of the cytokine may be restricted, and its usefulness nullified. Too strong of interactions between the stabilizing molecules may additionally limit the free stabilizing molecule from moving to the interface to shield the cytokine. Thus, as a broad design principle, it is essential for the cytokine and carrier protein to bind favorably when in aqueous solvent to a degree stronger than that of the stabilizing molecule with itself and a weaker than cytokine with receptor.

Protein absorption to interfaces has been studied extensively at a variety of interfaces (e.g., air–water,^27^, ^28^, ^29^, ^30^ oil–water,^31^, ^32^ organic–water,^33^, ^34^ solid–water,^35^ monolayer–water^29^, ^36^, ^37^) and with a variety of proteins (e.g., ovalbumin,^31^ lactalbumin,^34^ antibodies,^35^ lysozymes,^27^, ^29^, ^30^, ^34^, ^35^, ^36^, ^37^, ^38^, ^39^, ^40^ hydrophobins,^32^ insulin^28^). The amphipathic^34^, ^40^ nature of the proteins causes them to preferentially absorb at interfaces, often with specific preferred orientations. For instance, in one study, the amphipathic helices of a protein oriented parallel to the air–water interface, as similarly observed in our work.^40^ As previously described, we investigated the competing force between protein–protein interactions and protein–interface interactions that can govern stability and absorption of the proteins.^39^ Furthermore, polymers can also be protective at interfaces,^27^ but given that the presence of a polymer is held constant in our studies (not present in any computational simulations and present in all nanoparticle experiments), we focused our attention on the role of the protein interactions with the interface and each other.

In addition to stabilizing proteins like BSA, other molecules, such as polysaccharides (e.g., dextran) and sugars (e.g., trehalose), may be candidates for cytokine stabilization worth studying in future work.^1^ For example, the glycosaminoglycan heparin has been shown to bind strongly to hIL‐12 and mIL‐12 at the p40 subunit^41^ as well as protect hIL‐12 from loss of bioactivity.^23^, ^42^ Other cytokines, such as IFNγ, IL‐2,^41^ and IL‐6^43^ can also bind to heparin. In addition to binding strength of these molecules with the respective cytokines, the binding location is also crucial; as we show that hIL‐12 has a specific range of residues with the highest fluctuations, molecules that prohibit structural changes over that region will be best suited to protect it. Similarly, other cytokines likely have regions with a high propensity to unfold, and as such, different molecules may be preferable for stabilization of different cytokines due to binding location.

## 4 MATERIALS AND METHODS

### 4.1 MD simulations

The crystal structures of BSA and hIL‐12 were obtained from the Protein Data Bank (PDB) with codes 3 V03 and 1F45, respectively.^44^, ^45^ Missing residues in the structures were projected using MODELER or truncated at the unstructured end regions.^46^ For the interfacial simulations, Packmol was used to construct rectangular boxes,^47^ in which a single protein (either hIL‐12 or BSA) was placed at a specific distance and orientation relative to the bottom of the box (as described in the Section 2). The protein was also positioned to have approximately 1.3 nm of space on each side to prevent self‐interactions during subsequent MD simulations. Using Packmol,^47^ we then solvated the protein in pure water and the other half of the box with DCM, or vice versa, according to their bulk experimental densities, assumed to be 997 kg/m^3^ for water and 1322 kg/m^3^ for DCM. The same protocol was used to construct boxes of docked BSA‐hIL‐12 complexes,^44^ generated using ClusPro (see Section 2 for details),^21^ in the presence of a water/DCM interface.

For simulations of docked BSA‐hIL‐12 complexes generated using ClusPro (i.e., with no interface), as well as for simulations of hIL‐12 or BSA in pure solvent, cubic boxes were generated and solvated in GROMACS 2021,^48^ similarly ensuring at least 1.3 nm of space from the proteins and all box edges. In all, simulations ranged from 72,566 atoms for BSA in pure DCM to 1,694,642 atoms for hIL‐12 bound by four BSA molecules at a water/DCM interface.

In the MD simulations, BSA, hIL‐12, and neutralizing counterions were described using the OPLS‐AA force field, while the TIP4P force field was employed to model water.^49^, ^50^ Parameters for modeling DCM were primarily obtained from the OPLS‐AA force field, with DCM atomic charges taken from Caleman et al.^51^ All simulations used full periodic boundary conditions (PBC) in all directions, which, importantly, resulted in the presence of two water/DCM interfaces in each simulation. The protocol for simulations consisted of a series of two energy minimizations with increasingly small step sizes to ensure convergence, followed by two equilibration steps. The first equilibration step was conducted in the canonical (NVT) ensemble for 0.5 ns, with temperature maintained at 298.15 K using a velocity rescaling thermostat.^52^ Subsequently, equilibration in the isothermal‐isobaric (NPT) ensemble was performed for 1.0 ns, using the same thermostat and Berendsen barostat to maintain the system pressure at 1 bar.^53^

Finally, production simulations were similarly carried out in the NPT ensemble at the same temperature and pressure, with the same thermostat and Parrinello–Rahman barostat.^54^ A 2 fs time step was employed in all simulations, along with the LINCS (linear constraint solver) algorithm to constrain bonds between hydrogen and heavy atoms.^55^ Particle mesh Ewald summation was used to calculate long‐range electrostatic interactions, with a cutoff distance of 1.0 nm.^56^ Van der Waals interactions and neighbor lists also utilized a cutoff distance of 1.0 nm. Van der Waals interactions were shifted beyond this distance, and neighbor lists were updated every 10 steps.

During the MD simulations, slight mixing of the solvent phases occurred (e.g., some water was present in DCM and vice versa). However, the effects were minimal, as both the “pure” water and DCM phases consistently reached their bulk experimental densities far away from the protein and interfaces (Figure S13).

### 4.2 Simulation trajectory analysis

Multiple metrics were used to analyze the resulting MD simulation trajectories, including RMSD, RMSF, and interfacial distance calculations using PLUMED and Visual MD.^57^, ^58^ While not all simulations converged, as indicated by RMSD values that did not plateau over time, this outcome was expected due to the anticipated structural changes in the proteins in response to their environment, namely, the water/DCM interface. Both RMSD and RMSF calculations were performed in reference to Cα atoms in the initial structure used for the production MD simulations. RMSF calculations were conducted and averaged over the last 50 ns of each simulation trajectory. The distance between the protein and the interface was determined by calculating the locations of the COM of the proteins and comparing these to the computationally determined interface position at each simulation time point (see next section). Surface areas were calculated using Surface Racer.^59^

### 4.3 Interface location calculation

Alice Gast's *Physical Chemistry of Surfaces* ^60^ describes the Gibbs Dividing Surface to define the interface:

$$\left(c_{\mathrm{DCM}}\left(x_{a}\right)*\left[x_{b}-x_{a}\right]-\int_{x_{a}}^{x_{b}}C_{\mathrm{DCM}}\mathrm{dx}\right)+\left(\int_{x_{a}}^{x_{b}}C_{\text{Water}}\mathrm{dx}-C_{\text{Water}}\left(x_{a}\right)*\left[x_{b}-x_{a}\right]\right)\;=\left(\int_{x_{b}}^{x_{c}}C_{\mathrm{DCM}}\mathrm{dx}-C_{\mathrm{DCM}}\left(x_{c}\right)*\left[x_{c}-x_{b}\right]\right)+\left(C_{\text{Water}}\left(x_{c}\right)*\left[x_{c}-x_{b}\right]-\int_{x_{b}}^{x_{c}}C_{\text{Water}}\mathrm{dx}\right)$$

where $C_{i}$ is the concentration of each solvent, $x_{a}$ is the location at which the concentration begins to deviate from the bulk value, $x_{b}$ is location of the Gibbs Dividing Surface, and $x_{c}$ is the location at which the concentration returns to the bulk value. Figure S14a illustrates these parameters in the context of our system. However, as depicted in Figure S14b, the presence of a protein at the interface complicates this definition. With only one protein molecule present, the bulk cannot incorporate the concentration of the protein, leading to an ill‐defined deviation from bulk.

To ensure consistency in the definition of the interface regardless of the position of the protein, we identified DCM molecules within 5 Å of any water molecules, and vice versa. The number of molecules meeting these criteria was then plotted in separate histograms for water and DCM. The interfaces were determined as the midpoint between the peaks of these histograms (Figure S14c). This approach accurately describes where solvent species change, irrespective of the location of the protein in the simulation (Figure S14d), providing a reasonable approximation of the Gibbs Dividing Surface.^34^

### 4.4 Surface tension calculations

The average surface tension $\gamma \left(t\right)$ is computed in the GROMACS software suite as follows:

$$\gamma \left(t\right)=\frac{L_{Z}}{n}\left(P_{\mathrm{ZZ}}\left(t\right)-\frac{P_{\mathrm{XX}}\left(t\right)-P_{\mathrm{YY}}\left(t\right)}{2}\right)$$

where $L_{Z}$ represents the height of the simulation box in the Z direction, n indicates the number of surfaces, and $P_{\mathrm{ii}}\left(t\right)$ denotes the pressure component in the *i*‐direction at time t. This formula was adjusted for our simulations, which featured two interfaces (due to the PBC) aligned along the X direction:

$$\gamma \left(t\right)=\frac{L_{X}}{2\;\text{Interfaces}}\left(P_{\mathrm{XX}}\left(t\right)-\frac{P_{\mathrm{ZZ}}\left(t\right)-P_{\mathrm{YY}}\left(t\right)}{2}\right)$$

### 4.5 Particle preparation by double emulsion solvent evaporation

PLGA particles containing cytokines were formulated as previously described.^12^ Briefly, 8 mg PLGA (MW = 7–17 kDa) was dissolved in 400 μl DCM (Sigma Aldrich) to form the organic phase. Five micrograms hIL‐12 (Peprotech) dissolved in deionized water (DIW) at 1 μg/μl was added to 75 μl PBS in a low‐protein binding microcentrifuge tube with a final concentration of 0–0.1 wt.% BSA (Sigma Aldrich). This aqueous phase was either incubated with 400 rpm shaking at room temperature for 10 min or transferred directly to the organic phase without mixing. The mixture of 1:5 by volume aqueous to organic phase was rapidly vortexed for 20 s and added to a bath sonicator for 3 min to form the primary emulsion. Dropwise, the primary emulsion was added to 5 ml of 1.5 wt.% PVA (Sigma Aldrich) solution in a scintillation vial stirring at 1200 rpm. The PVA solution was made by measuring 1.5 g PVA, filling to 100 ml with DIW, and mixing in a round bottom flask for 3 h or until crystal dissolution at 85°C with an attached condenser. The secondary emulsion was then formed in the PVA aqueous phase by sonicating the mixture with a microprobe sonicator (Fisher Scientific) at 70% magnitude, pulsing for 20 s on, 10 s off, and 20 s on. DCM was evaporated overnight by stirring at 300 rpm in an open container, resulting in a suspension of hIL‐12‐loaded PLGA nanoparticles.

### 4.6 Particle characterization

Particles in DIW were analyzed by DLS (Anton Paar) to measure size and zeta potential. Measurements were performed at room temperature in Omega cuvettes with automatic stopping criteria to ensure an adequate number of measurements were taken. Size measurements were also confirmed with SEM. Imaging was performed in secondary electron imaging mode with high vacuum at an accelerating voltage of 15,000 V (Hitachi SU3500). Particles were deposited on a clean silicon wafer and coated in 10 nm platinum using a sputter coater (Cressington 108auto) for imaging.

### 4.7 Cytokine release experiments

After evaporation of DCM from the particles, the suspension of particles was split into low protein binding microcentrifuge tubes and washed twice in fresh DIW by centrifugation at 12,000xg for 10 min to remove PVA and prematurely released hIL‐12. Particle aliquots were resuspended in 300 μl PBS for release, and at each timepoint, particles were centrifuged at 12,000xg for 5 min, the supernatant collected, and particles resuspended in 300 μl fresh PBS. BSA was added to all collected supernatants to reach 0.1% solutions to prevent loss of hIL‐12 when freezing. Supernatants were frozen immediately at −20°C until analysis.

### 4.8 ELISA for hIL‐12 quantification

Supernatant from hIL‐12 release studies was stored in low protein binding microcentrifuge tubes at −20°C until use. All samples were measured within a month of freezing. When ready to use, samples were thawed at room temperature and analyzed on a human IL‐12p70 ELISA kit (STEMCELL) or murine IL‐12p70 ELISA kit (Invitrogen) according to manufacturer instructions.

### 4.9 PBMC isolation

With donor consent, whole blood was obtained from a healthy deidentified female donor by venipuncture and collected in K2 EDTA‐coated vacuum tubes (Thermo Fisher) to prevent coagulation, following an approved protocol from the institutional review board at the University of Colorado Boulder (22‐0175) and in accordance with the ethical standards of the responsible committee on human experimentation (institutional and national) and the Helsinki Declaration of 1975, revised in 2008. Blood was separated using Ficoll‐Paque PLUS (Cytivia) according to manufacturer instructions to isolate PBMCs. Cells were resuspended in RPMI 1640 medium (Thermo Fisher) supplemented with 10 vol.% heat‐inactivated FBS and 1 vol.% penicillin–streptomycin (VWR) and incubated at 37°C and 5% CO_2_.

### 4.10 hIL‐12 bioactivity assay

IFNγ secretion from PBMCs was used as a measure of hIL‐12 activity.^23^, ^42^ PBMCs were seeded into a 96‐well plate at 0.2 × 10^6^ cells/well in 200 μl RPMI 1640 medium with 10 μl/ml ImmunoCult™ Human CD3/CD28/CD2 T Cell Activator (STEMCELL). After 24 h of incubation, cells were collected from wells into microcentrifuge tubes and pelleted at 400xg for 10 min. Media was aspirated and replaced with fresh RPMI 1640 and hIL‐12 or particles according to the experimental condition. After an additional 24 h of incubation, cells were collected from wells and pelleted as before, and the supernatant was saved in low protein binding microcentrifuge tubes. Cell media containing IFNγ was frozen until use, at which point it was measured using a human IFNγ ELISA kit (Invitrogen) according to manufacturer instructions. While donor biological variables (sex, ethnicity, age) may have impacted the cellular activity and response to activator and hIL‐12, all IFNγ secretion data was normalized to the control condition and therefore isolated relative trends only rather than depending on the donor.

### 4.11 Statistical methods

Unless otherwise indicated, all experimental data is shown as average ± standard deviation. Unpaired *t* tests or one‐way ANOVA were used for determination of significance with the following cutoff points: *p < 0.05, **p < 0.01, ***p < 0.001.

## 5 CONCLUSIONS

Particle‐based delivery of unstable biologics, such as cytokines, chemokines, enzymes, and growth factors, has the potential to improve therapeutic efficacy through longer circulation times, sustained release, and additional opportunities for functionalization. We sought to elucidate the mechanisms by which the cytokine IL‐12 can be stabilized in polymer particles using carrier proteins due to its clinical relevance for cancer therapy, among other diseases. Using MD, we demonstrated the instability of hIL‐12 at the interface of DCM and water formed during the primary emulsification step of particle formulation. Furthermore, the RMSF of hIL‐12 at the interface indicated that there was a key region of instability in the p40 subunit that may be responsible for significant deviations observed in hIL‐12. However, when BSA molecules bound to an hIL‐12 molecule in their most common configurations, hIL‐12 structural changes caused by the interface were dampened. Additionally, by performing controlled release studies from particles formulated with hIL‐12 and BSA, we identified that excess, unbound BSA also contributes to the protection of hIL‐12. We therefore propose that hIL‐12 can be stabilized in particles formulated with double emulsification by both (i) interfacial crowding by excess BSA, and (ii) protein–protein binding between molecules to prevent unfolding. These insights, and the approaches used herein, may enable the screening of additional cytokine‐stabilizing agent pairings for the rational design and formulation of improved drug delivery vehicles.

## AUTHOR CONTRIBUTIONS

**Emily R. Rhodes:** Conceptualization; investigation; writing – original draft; writing – review and editing; formal analysis. **Nicole B. Day:** Conceptualization; investigation; writing – original draft; writing – review and editing; formal analysis. **Emma C. Aldrich:** Conceptualization; investigation; writing – original draft; writing – review and editing; formal analysis. **C. Wyatt Shields IV:** Conceptualization; writing – review and editing; funding acquisition; project administration. **Kayla G. Sprenger:** Conceptualization; funding acquisition; writing – review and editing; project administration.

## CONFLICT OF INTEREST STATEMENT

The authors have no conflicts of interest to declare.

## Supporting information


**DATA S1:** Supplementary Information.

**SUPPLEMENTARY VIDEO 1:** Water, DCM and BSA atomistic density over time plotted against the orthogonal dimension of the simulation box for an example simulation.

**SUPPLEMENTARY VIDEO 2:** Water, DCM and hIL‐12 atomistic density over time plotted against the orthogonal dimension of the simulation box for an example simulation.

## Data Availability

The data that support the findings of this study are available from the corresponding author upon reasonable request.
