# Supplementary material for: Elucidating the role of carrier proteins in cytokine stabilization within double emulsion‐based polymeric nanoparticles
Source: Bioeng Transl Med. 2024 Sep 5;10(1):e10722. doi: 10.1002/btm2.10722 (PMC11711224; doi:10.1002/btm2.10722)
Supplement: Supplementary file 1 — DATA S1: Supplementary Information. [file BTM2-10-e10722-s001.pdf]

Supporting Information for

Elucidating the Role of Carrier Proteins in Cytokine Stabilization within Double Emulsion-Based Polymeric Nanoparticles

Emily R. Rhodes[1,*], Nicole B. Day[1,*], Emma C. Aldrich[1],
C. Wyatt Shields IV[1,2+], Kayla G. Sprenger[1,2+]

1. Department of Chemical and Biological Engineering, University of Colorado Boulder, Boulder, Colorado 80303, United States

2. Biomedical Engineering Program, University of Colorado Boulder, Boulder, Colorado 80303, United States

[*]Authors contributed equally; [+]Co-corresponding authors: Charles.Shields@colorado.edu and Kayla.Sprenger@colorado.edu

This PDF file includes:

I. Supplementary Materials

Table S1. Interface residue properties for each orientation of BSA and hIL-12.

II. Supplementary Figures

Figure S1. Particle size distributions.
Figure S2. Final snapshots from Supplemental Videos 1 and 2 of density over time.
Figure S3. Distance of BSA and hIL-12 from the interface, separated by starting solvent.
Figure S4. Characterization of hydrogen bonds formed between BSA and hIL-12 with water.
Figure S5. Protein interfacial alignment results from surface tension.
Figure S6. Protein density width in simulations colored by simulation time.
Figure S7. RMSD over time.
Figure S8. Proximity to the interface for each protein residue across all orientations.
Figure S9. Average RMSD of simulations with increasing amounts of BSA.
Figure S10. RMSF of simulations with increasing amounts of BSA.
Figure S11. Sequence alignment of murine and human IL-12.
Figure S12. Structural alignment of human and murine IL-12.
Figure S13. Density of bulk phases away from the protein and interfaces.
Figure S14. Gibbs Dividing Surface diagrams compared with computed interface definition.

I. SUPPLEMENTARY MATERIALS

Table S1. Interface residue properties for each orientation of BSA and hIL-12.

	**BSA**		**hIL-12**	
	**Number of Close Residues**	**Interface Accessible Surface Area (Å^2)**	**Number of Close Residues**	**Interface Accessible Surface Area (Å^2)**
**Red**	24	1417.3	11	1511.5
**Blue**	23	1202.0	6	635.2
**Green**	17	1031.8	21	1547.3
**Yellow**	53	3084.8	47	2990.3
**Orange**	35	2376.7	25	1875.4
**Purple**	29	2140.1	40	3256.1

II. SUPPLEMENTARY FIGURES

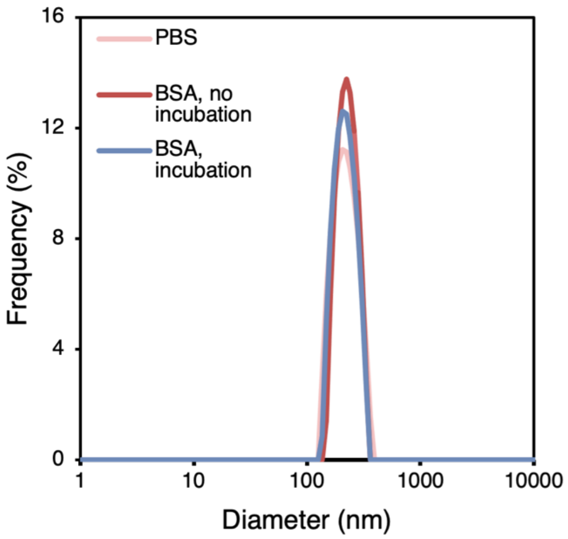

Figure S1. Particle size distributions. Particles used in hIL-12 release experiments were analyzed using DLS.

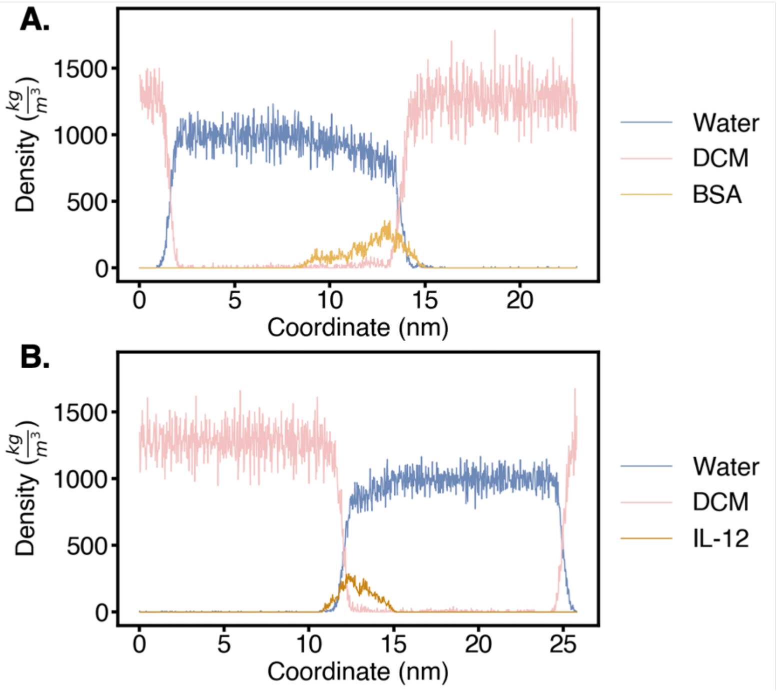

Figure S2. Final snapshots from Supplemental Videos 1 and 2 of density over time. A) BSA and B) hIL-12 navigate across the DCM/water interface–localizing primarily on the water side of the interface, though their structures fully span the interface.

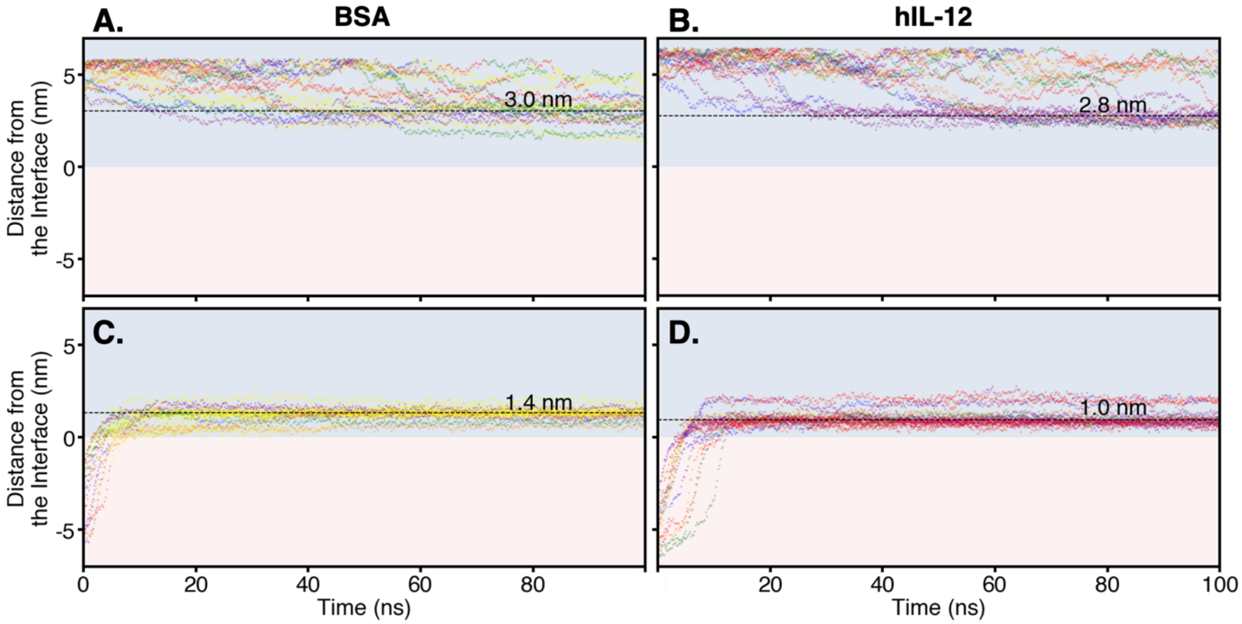

Figure S3. Distance of BSA and hIL-12 from the interface, separated by starting solvent. BSA and hIL-12 localize further from the interface when initiating in water (A, B, respectively) versus in DCM (C, D, respectively), as indicated by their median final distances (dashed lines).

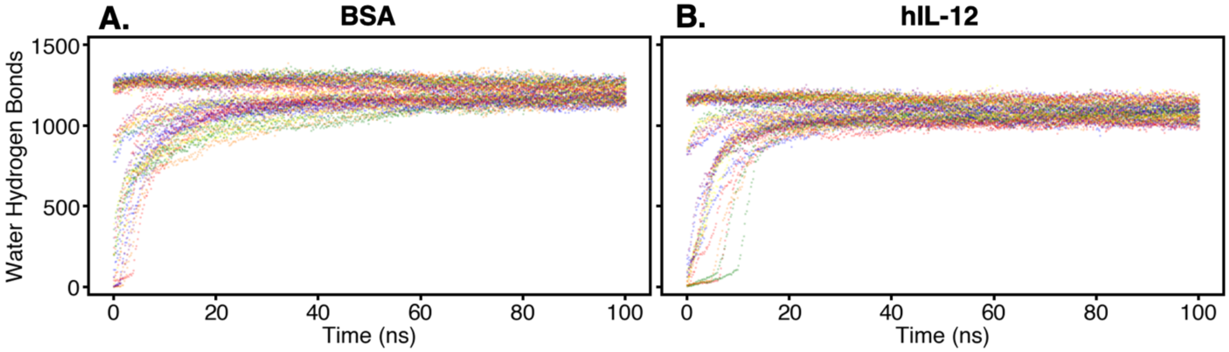

Figure S4. Characterization of hydrogen bonds formed between BSA and hIL-12 with water. The number of hydrogen bonds A) BSA and B) hIL-12 form with water initially increases and then remains constant with time, independent of the starting position of the protein. Hydrogen bonds occurring in the pure DCM phase were facilitated by water molecules that had spontaneously partitioned into the DCM phase during the simulations.

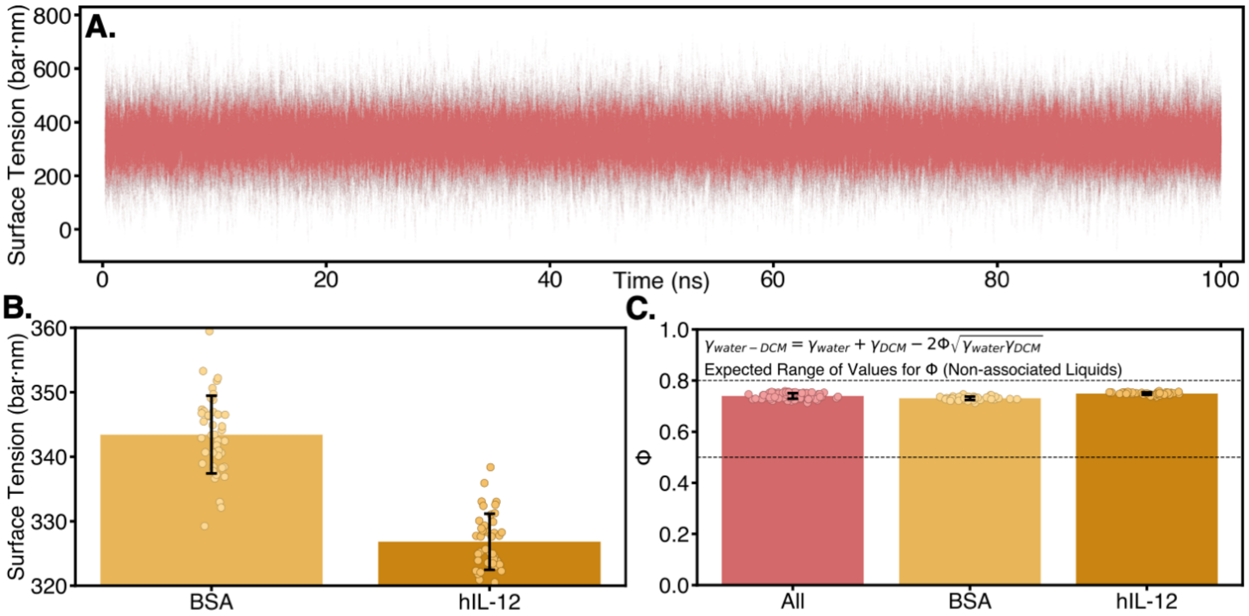

Figure S5. Protein interfacial alignment results from surface tension. A) Surface tension, calculated as a function of pressure in the MD simulations, remains constant over the course of all BSA and hIL-12 simulations. B) The surface tension for BSA and hIL-12 averaged for the whole simulations with standard deviations shown across simulations. C) The phi (Φ) value, used to calculate the surface tension, is within the expected range for non-associated liquids.

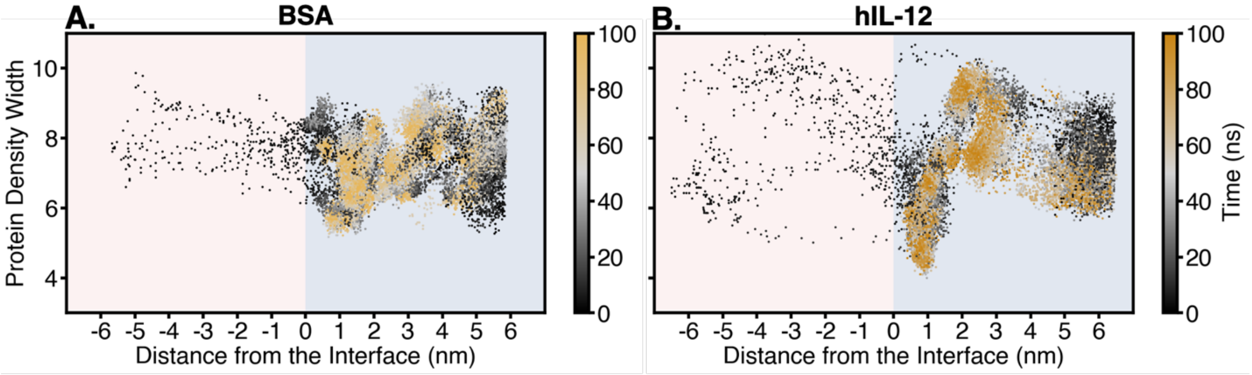

Figure S6. Protein density width in simulations colored by simulation time. Protein density width of A) BSA and B) hIL-12 as a function of time.

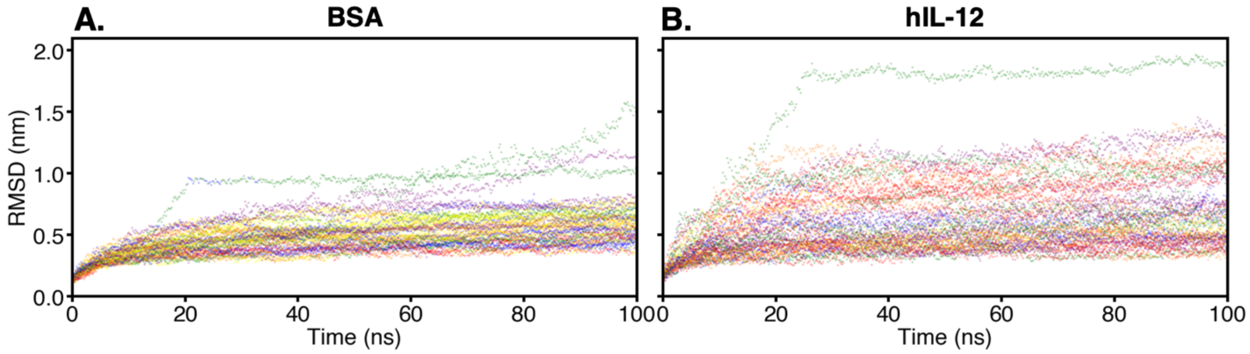

Figure S7. RMSD over time. Over the course of the simulations with an interface, the RMSD of both A) BSA and B) hIL-12 increases relative to their respective crystal structures. hIL-12 shows a larger increase in RMSD than BSA.

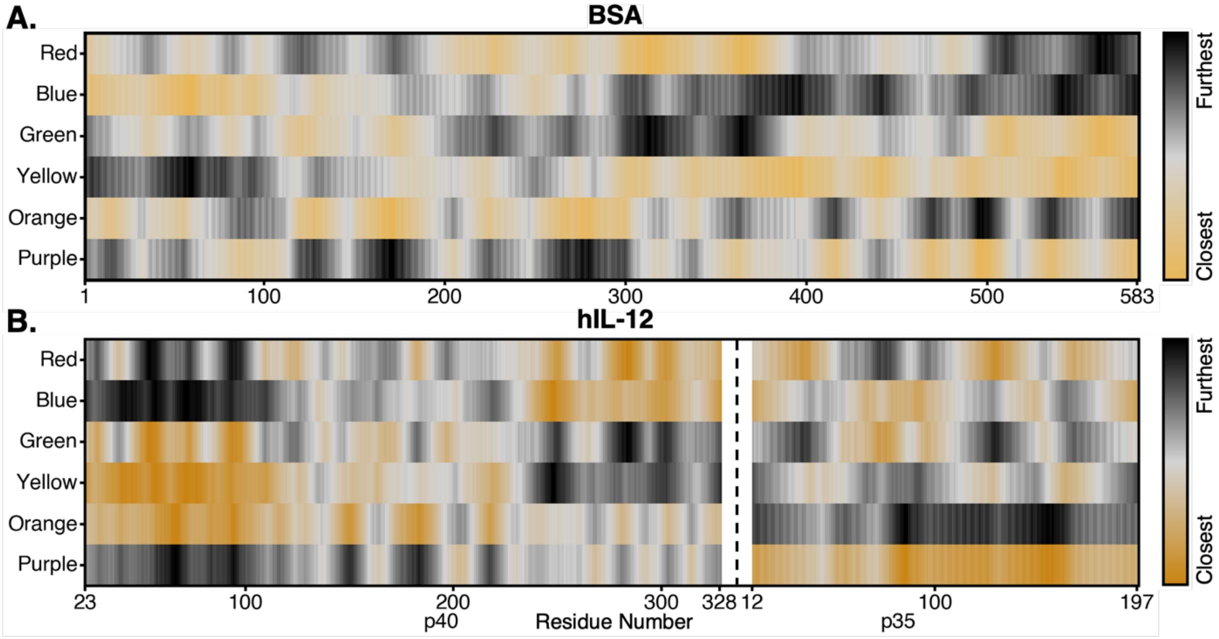

Figure S8. Proximity to the interface for each protein residue across all orientations. A) BSA and B) hIL-12 distance from the interface with each orientation indicated by color.

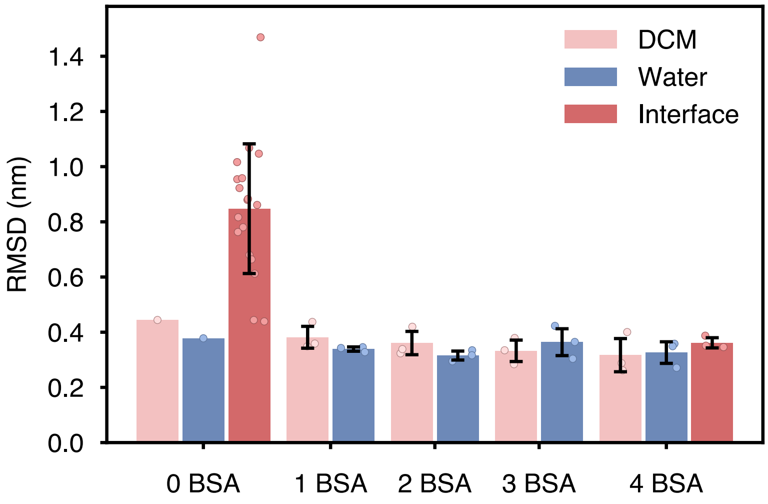

Figure S9. Average RMSD of simulations with increasing amounts of BSA. N = 1 (0 BSA, DCM and water); N = 3 ± SD (0 BSA, Interface; 1-4 BSA), N = 42 ± SD (4 BSA, Interface).

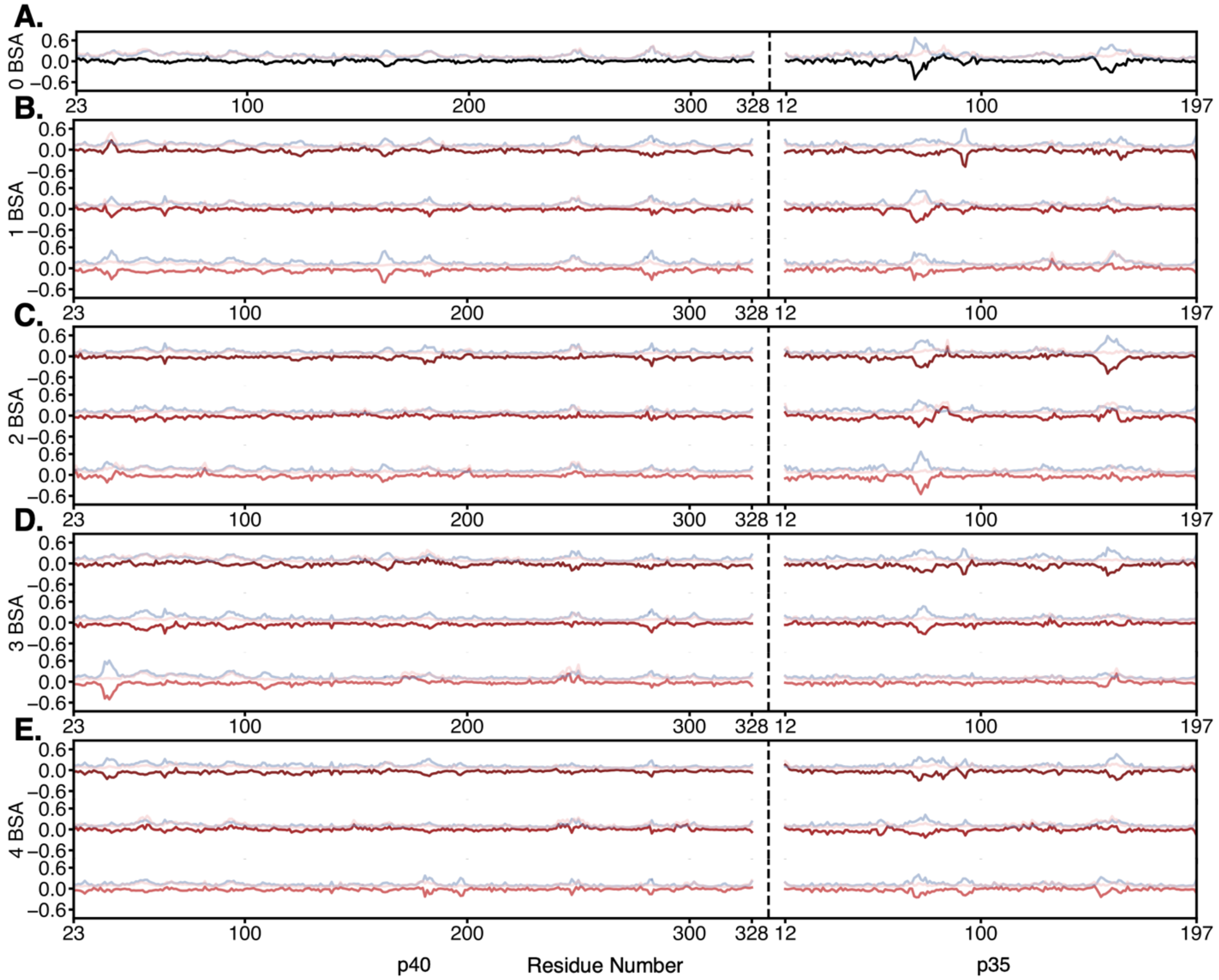

Figure S10. RMSF of simulations with increasing amounts of BSA. A) The RMSF of plain hIL-12 in water (blue line) and DCM (pink line), with the difference indicated by the black line. B-E) Increasing amounts of BSA added to hIL-12 and simulated (in triplicate) in water (blue line) and DCM (pink line) with the difference indicated by lines in shades of red corresponding to each replicate.

```
A. tr|O60595|O60595_HUMAN      MWPPGSASQPPPSPAAATGLHPAARPVSLQCRLSMCPARSLLLVATLVLLDHLSLARNLP      60
   tr|Q9QUT1|Q9QUT1_MOUSE      ----------------------------------MCQSRYLLFLATLALLNHLSLARVIP      26
                                                                 ** :* **::***.**:****** :*

   tr|O60595|O60595_HUMAN      VATPDPGMFPCLHHSQNLLRAVSNMLQKARQTLEFYPCTSEEIDHEDITKDKTSTVEACL      120
   tr|Q9QUT1|Q9QUT1_MOUSE      VSGP----ARCLSQSRNLLKTTDDMVKTAREKLKHYSCTAEDIDHEDITRDQTSTLKTCL      82
                               *: *      ** :*:***::..:*::.**:.*:.* **:*:*******:*:***:::**

   tr|O60595|O60595_HUMAN      PLELTKNESCLNSRETSFITNGSCLASRKTSFMMALCLSSIYEDLKMYQVEFKTMNAKLL      180
   tr|Q9QUT1|Q9QUT1_MOUSE      PLELHKNESCLATRETSSTTVSKH---SKGSFPMTDS-----------------------      116
                               **** ****** :****  * ..     * ** *: .

   tr|O60595|O60595_HUMAN      MDPKRQIFLDQNMLAVIDELMQALNFNSETVPQKSSLEEPDFYKTKIKLCILLHAFRIRA      240
   tr|Q9QUT1|Q9QUT1_MOUSE      ------------------------------------------------------------      116

   tr|O60595|O60595_HUMAN      VTIDRVMSYLNAS      253
   tr|Q9QUT1|Q9QUT1_MOUSE      -------------      116
```

```
B. sp|P29460|IL12B_HUMAN      MCHQQLVISWFSLVFLASPLVAIWELKKDVYVVELDWYPDAPGEMVVLTCDTPEEDGITW 60
   sp|P43432|IL12B_MOUSE      MCPQKLTISWFAIVLLVSPLMAMWELEKDVYVVEVDWTPDAPGETVNLTCDTPEEDDITW 60
                              ** *:*.****::*:*.***:*:***:*******:** ****** * *********.***

   sp|P29460|IL12B_HUMAN      TLDQSSEVLGSGKTLTIQVKEFGDAGQYTCHKGGEVLSHSLLLLHKKEDGIWSTDILKDQ 120
   sp|P43432|IL12B_MOUSE      TSDQRHGVIGSGKTLTITVKEFLDAGQYTCHKGGETLSHSHLLLHKKENGIWSTEILKNF 120
                              * **   *:******** **** ************.**** *******:*****:***:

   sp|P29460|IL12B_HUMAN      KEPKNKTFLRCEAKNYSGRFTCWWLTTISTDLTFSVKSSRGSSDPQGVTCGAATLSAERV 180
   sp|P43432|IL12B_MOUSE      ---KNKTFLKCEAPNYSGRFTCSWLVQRNMDLKFNIKSSSSSPDSRAVTCGMASLSAEKV 177
                                 ******:*** ******** **.  . **.*.:*** .* * :.**** *:****:*

   sp|P29460|IL12B_HUMAN      RGDNKEYE-YSVECQEDSACPAAEESLPIEVMVDAVHKLKYENYTSSFFIRDIIKPDPPK 239
   sp|P43432|IL12B_MOUSE      TLDQRDYEKYSVSCQEDVTCPTAEETLPIELALEARQQNKYENYSTSFFIRDIIKPDPPK 237
                                *:::** ***.**** :**:***:****: ::* :: *****::**************

   sp|P29460|IL12B_HUMAN      NLQLKPLKNSRQVEVSWEYPDTWSTPHSYFSLTFCVQVQGKSKREKK--------DRVFT 291
   sp|P43432|IL12B_MOUSE      NLQMKPLKNS-QVEVSWEYPDSWSTPHSYFSLKFFVRIQRKKEKMKETEEGCNQKGAFLV 296
                              ***:****** **********:**********.* *::* *.:: *:        . .:.

   sp|P29460|IL12B_HUMAN      DKTSATVICRKNASISVRAQDRYYSSSWSEWASVPCS---      328
   sp|P43432|IL12B_MOUSE      EKTSTEVQC-KGGNVCVQAQDRYYNSSCSKWACVPCRVRS      335
                              :***: * * *...:.*:******.** *:**.***
```

Figure S11. Sequence alignment of murine and human IL-12. A) IL-12 alpha and B) beta subunits. Uniprot accessions for alpha subunits are O60595 (same sequence as P294959) and Q9QUTI for human and mice, respectively. Uniprot accessions for beta subunits are P29460 and P43432 for human and mice, respectively. PDB 1F45 (used in computational simulations) is comprised of Uniprot P29459 and Uniprot P29460.

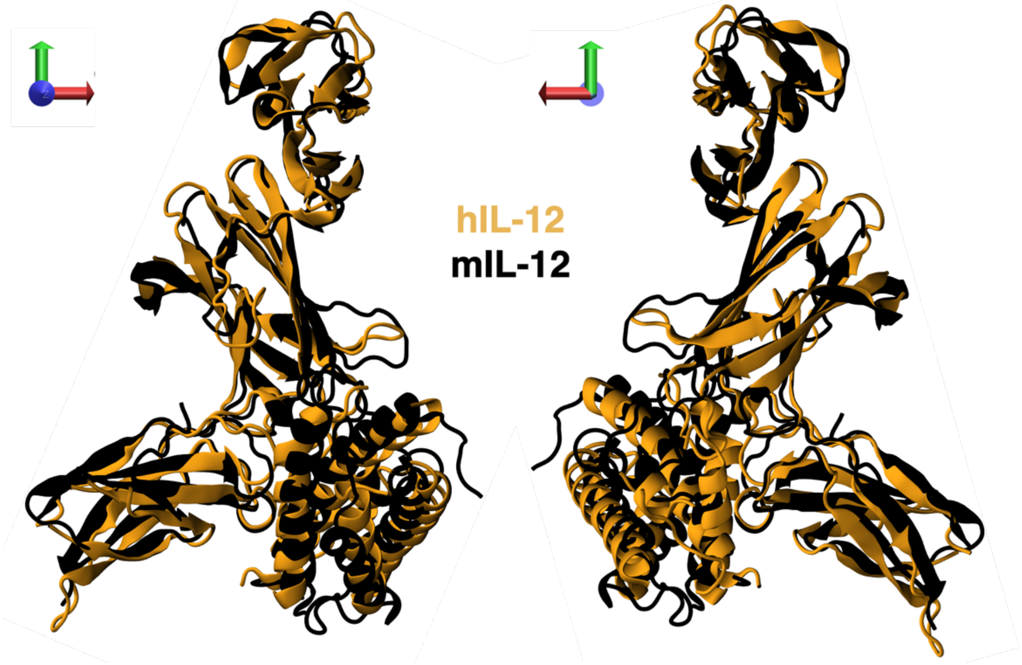

Figure S12. Structural alignment of human and murine IL-12. Human IL-12, PDB: 1F45 (gold), and murine IL-12, PDB: 8CR6 (black), aligned by ClustalW.

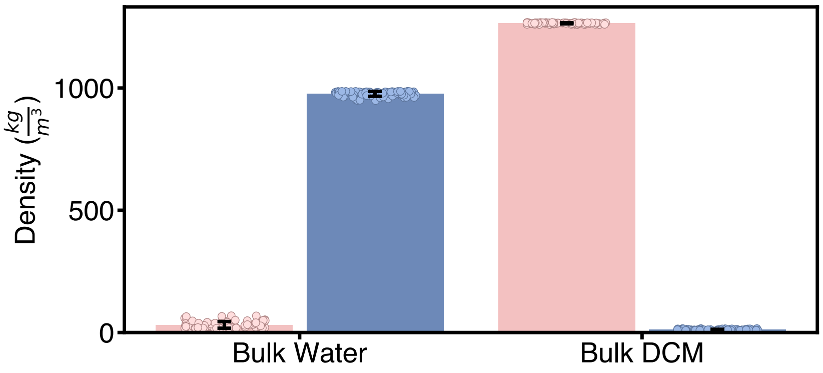

Figure S13. Density of bulk phases away from the protein and interfaces. Bulk water contains mostly water (blue) and a small number of DCM molecules (pink) while bulk DCM contains mostly DCM (pink) and a small number of water molecules (blue) due to a small amount of mixing between solvents.

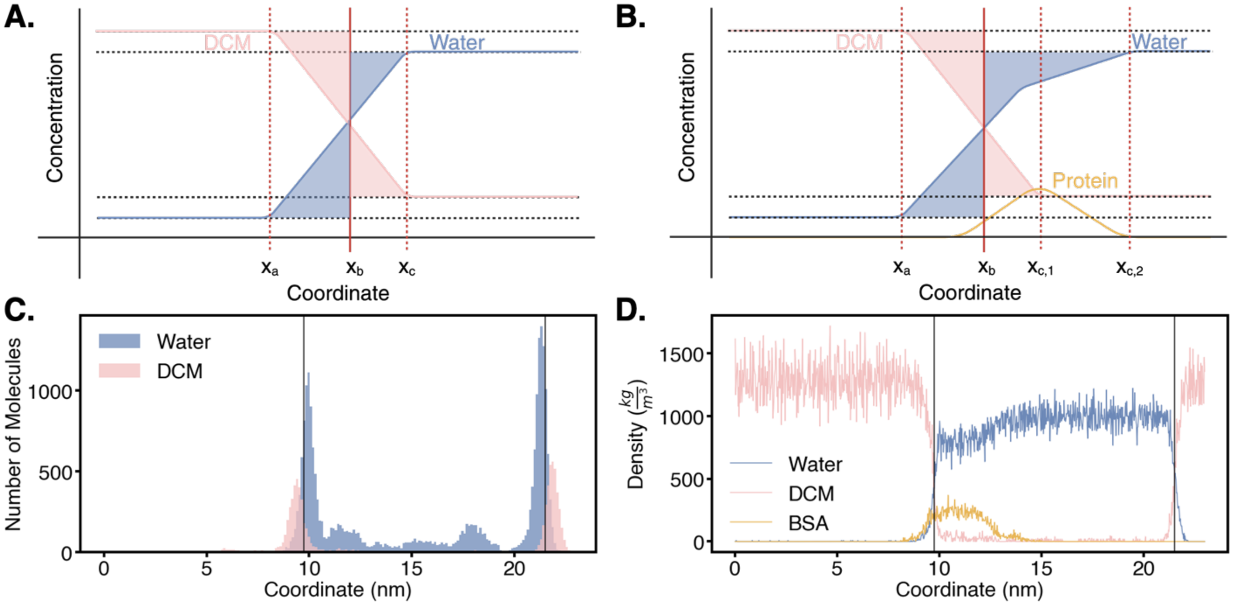

Figure S14. Gibbs Dividing Surface diagrams compared with computed interface definition. A) The Gibbs Dividing Surface is described (x_b) as where the change in concentration, shown by the shaded regions, is equal on either side of the interface. B) In our simulations, the definition of the Gibbs Dividing Surface is disrupted by the presence of the protein at the interface. C) In practice, the dividing surface can be accurately defined by identifying the point at which molecules of one solvent are in close proximity to molecules of another solvent. D) In our simulations, the Gibbs Dividing Surfaces match closely with the changes in density of the solvent.
